# Nonlinear mode saturation in a U-shaped micro-resonator

**DOI:** 10.1038/s41598-022-14657-1

**Published:** 2022-06-21

**Authors:** Rodrigo T. Rocha, Mohammad I. Younis

**Affiliations:** 1 Physical Sciences and Engineering Division, King Abdullah University of Science and Technology–KAUST, Thuwal, 23955 Saudi Arabia; 2 Department of Mechanical Engineering, State University of New York, Binghamton, NY 13902 United States

**Keywords:** Engineering, Nanoscience and technology

## Abstract

Saturation is an intriguing phenomenon that has captured the attention of scientists since the time of Froude when he reported it for ship motion in the mid of the nineteenth century. This work presents the demonstration and a comprehensive study of the nonlinear saturation phenomenon on a compound micromachined structure of U-shape (micro portal frame). The frame is designed and fabricated as a multi-input and multi-output device for actuating the 1st (sway) and 2nd (symmetric) in-plane vibration modes. Geometric nonlinearities along with the softening effect of the electrostatic force present the necessary conditions for the activation of a 2:1 internal (auto-parametric) resonance between the 1st and 2nd modes. Experimental data complemented with analytical simulations are obtained showing the internal resonance and the saturation phenomenon. These results are promising for further exploration of such compound structures and for further in-depth studies of the saturation phenomenon on a variety of other systems and applications.

## Introduction

Micro- and Nano-electromechanical systems (MEMS/NEMS) have been explored and implemented for many domestic, industrial, health care, and military applications such as gas sensors, Radio Frequency RF switches, inertial switches, mass and gas detectors, and logic devices^1–7^.

Micro/nano structures form the backbone of a main category of devices, resonant sensors, in which a structure is driven to vibrate at resonance. When subjected to a physical or chemical stimulus, its shift in resonance frequency is used to measure and reveal the stimulus effect. The most commonly employed micro/nano structures as resonators are cantilever and clamped–clamped beams due to the ease of fabrication and accessing their modes of vibration. These structures have shown various nonlinear behaviors when undergoing large deformations and when subjected to multiple forms of excitations^8–12^.

Among the fascinating nonlinear phenomena is nonlinear mode saturation, which has captured the attention of scientists since the time of Froude when he reported it for ship motion in the mid of the nineteenth century^13^. Since then, considerable focus has been dedicated to this phenomenon and the nonlinear interaction among modes of vibration in various systems. This phenomenon is associated with internal or auto-parametric resonance involving nonlinear coupling and energy transfer among various modes of vibrations. It refers to the saturation of a vibration mode when it is externally excited around resonance, and the injected surplus energy is transferred to another unexcited mode^14^. It occurs under a 2:1 internal resonance between two modes of vibration due to the quadratic nonlinear coupling.

Experimental data of the phenomenon remain scarce due to the difficulty of controlling stiffness, damping, and other parameters that form the necessary conditions for its realization. With the advancements of fundamental research on Micro and Nano-electromechanical systems (MEMS/NEMS), which are viewed as ideal platforms for probing physical phenomena, the recent decade has shown several demonstrations of nonlinear mode coupling in micro- and nano-structures; however, most of these studies were based on simple structures, such as beams and diaphragms.

Internal resonances and saturation phenomenon have been observed and studied for MEMS resonators^15–18^. Recently, this concept has been proposed for frequency stabilization and noise reduction in MEMS resonators^19–23^, which is shown to be promising for frequency fluctuation reduction for lower vibration modes through the excitation of higher-order modes^23^.

Most of the reported works have been focused on single and simple structures. Compound structures (composed of more than one connected structural element) have received less attention. Examples of these are L-shaped beams, U-shaped beams (portal frames), and T-shaped beams. At the large scale, such structures have been shown theoretically^24,25^ and experimentally^26^ to possess interesting nonlinear behaviors, including internal resonance and the saturation phenomenon.

Micromachined compound structures have been utilized, however, mainly for static, linear, and limited range applications. U-shaped structures have been mainly used as multiple inputs and multiple outputs (MIMO) structures offering various capabilities and possibilities such as cold-hot actuators, energy harvesting devices, and multifunctional logic operations^27–29^.

In a previous work, the nonlinear softening behavior and frequency shift due to electrostatic actuation were investigated for a U-shaped micromachined structure^30^. The work showed the dominance of geometric quadratic nonlinearities and the frequency shift for higher-order modes, shedding light on MIMO devices and their potential applications.

The dynamic behavior of U-shaped micromachined structures, especially its nonlinear behavior, has not received adequate attention. In this work, we present an investigation into the nonlinear dynamics of a U-shaped micro-resonator portal frame. The device is designed to have a 2:1 internal resonance between the 1st and 2nd in-plane modes, at which the saturation phenomenon is also demonstrated. The investigation of the phenomenon is carried out through experimental data along with the mathematical model.

## Material and methods

### Fabricated device

**Figure 1:**
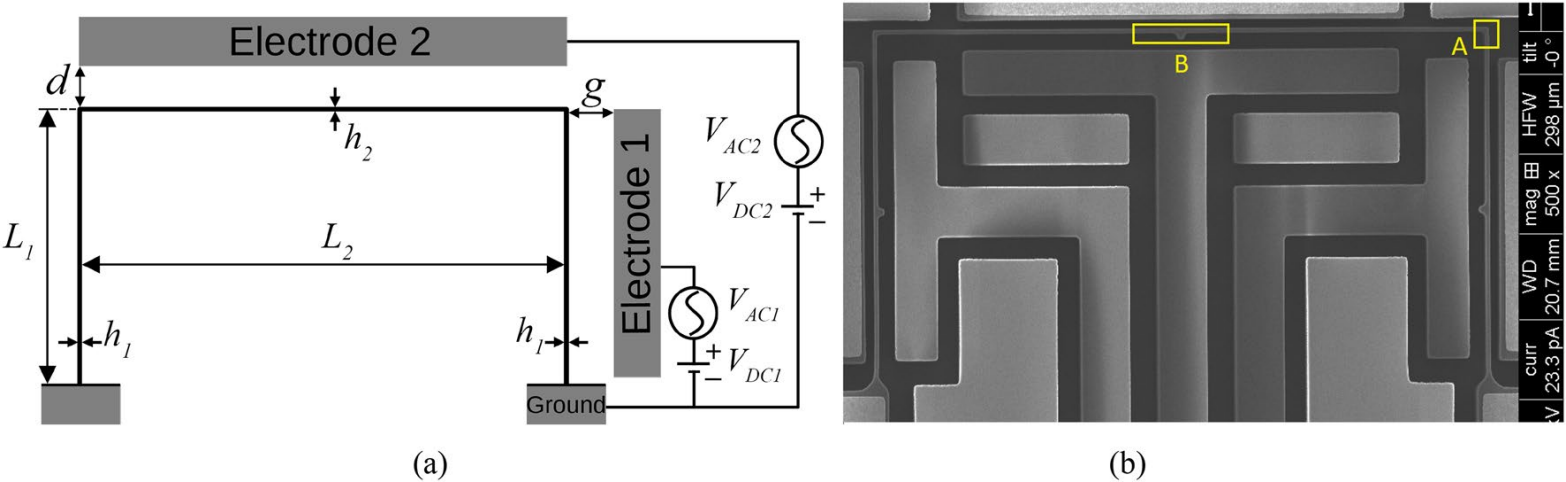
(**a**) Schematic of the micro portal frame with the actuation electrodes; (**b**) Scanning Electron Microscope (SEM) picture of the top view of the fabricated MEMS portal frame made of silicon. Squares A and B are the camera’s targets of the experimental equipment to measure the 1st and 2nd modes, respectively. Square A is set on the end of a column, while square B is set on the mid-span of the supported beam.

The micro portal frame shown in Fig. 1a,b is fabricated by the SOIMUMPs process of MEMSCAP^31^. The nominal features of the microstructure are as follows. It has a Young Modulus of *E* = 136GPa, and consists of the short beam elements (columns) of length *L*_1_ = 133.1 µm, the long beam element (supported beam) of length *L*_2_ = 240 µm, and the out-of-plane dimension of 25 µm. The beam and columns widths are measured respectively as *h*_2_ = 1.73 µm and *h*_1_ = 1.6 µm. In addition, the microstructure is subjected to an electrostatic force through two electrodes, one on top of the supported beam (Electrode 2 (E2)) by a DC actuation voltage *V*_*DC2*_ and an AC harmonic voltage *V*_*AC2*_, and the other on the column (Electrode 1 (E1)) by a *V*_*DC1*_ and a *V*_*AC1*_, with the distance between the supported beam and columns from their electrodes of *d* = 3.55 µm and *g* = 3.51 µm, respectively.

### Mathematical model for the nonlinear dynamic analysis

**Figure 2:**
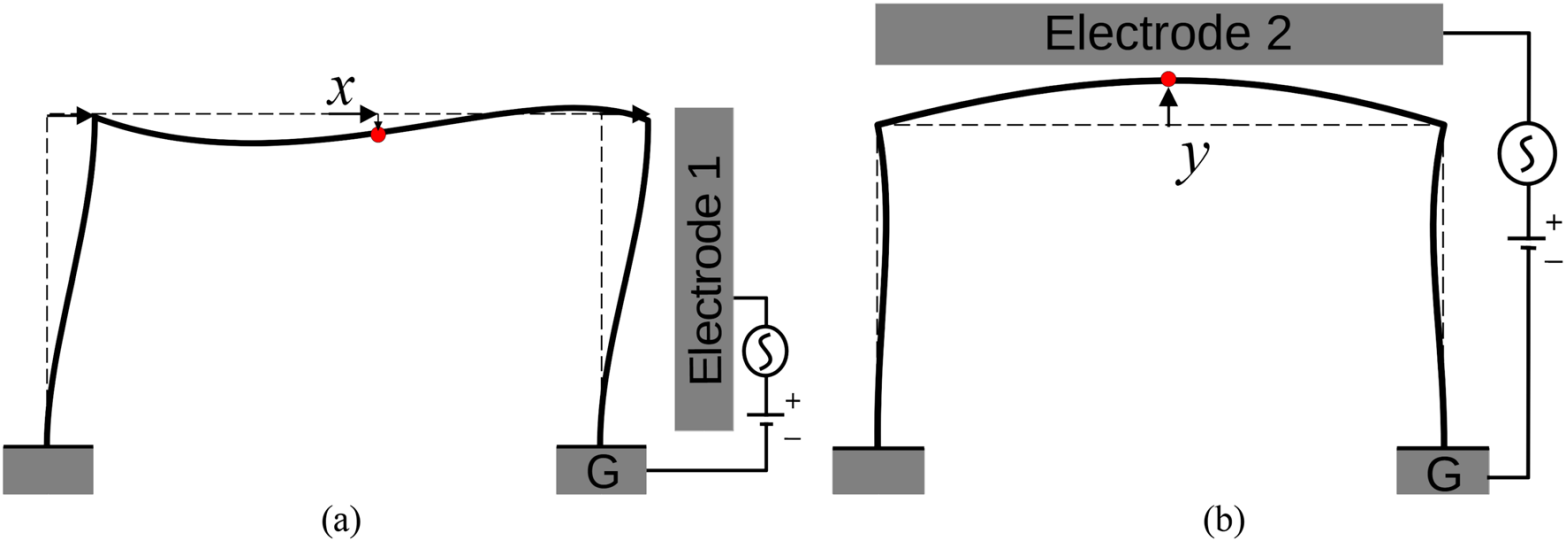
Schematic for (**a**) the 1st vibration mode under the actuation of E1, and (**b**) the 2nd vibration mode under the actuation of E2, of the micro portal frame model. Note that the 1st and 2nd modes are in the orthogonal direction, where the 1st mode motion is dominantly in the X-direction, while in the 2nd mode, the structure moves mainly in the Y-direction. The dashed line represents the microstructure at rest, and the generalized coordinates *x* and *y* set in the mid-span of the supported beam denote the motion at the position of the colored circle.

A mathematical model is developed to simulate the 1st and 2nd modal responses of the system, as illustrated in Fig. 2a,b, respectively. The generalized coordinates *x* and *y* represent the sway mode (1st mode) and the first symmetric bending mode (2nd mode) of the portal frame, respectively, and are set in the mid-span of the supported beam, square B of Fig. 1b. The shortening due to the bending of the columns and beam is the source of quadratic geometric nonlinearities of the structure.

The dimensionless equations of motion of the 1st and 2nd modes of the portal frame subjected to the electrostatic force of electrodes E1 and E2 are expressed, respectively, as

1 $$\ddot{x} + \mu_{1} \dot{x} + x + k_{31} x^{3} + \alpha_{1} xy + \delta_{1} \left( {x\dot{x}^{2} + x^{2} \ddot{x}} \right) = \beta_{1} \frac{{\left( {V_{DC1} + V_{AC1} \cos \Omega_{1} \tau } \right)^{2} }}{{\left( {1 - x} \right)^{2} }}$$

2 $$\ddot{y} + \mu_{2} \dot{y} + \omega_{n2}^{2} y + k_{32} y^{3} + \alpha_{2} x^{2} + \delta_{2} \left( {y\dot{y}^{2} + y^{2} \ddot{y}} \right) = \beta_{2} \frac{{\left( {V_{DC2} + V_{AC2} \cos \Omega_{2} \tau } \right)^{2} }}{{\left( {1 - y} \right)^{2} }}$$

where the dimensionless coefficients are defined as in the Supplementary Material. The complete derivation of the equations of motion of the micro portal frame is detailed in the Supplementary Material. Note that Eqs. (1) and (2) are weakly coupled through the quadratic nonlinearities involving the coefficients α_1_ and α_2_, which is one of the conditions for the activation of the saturation phenomenon. Because the actual dimensions and properties of the device vary from the nominal values due to imperfections and non-uniformity in fabrications, and to match the natural frequencies between experiments and simulations, we fit the dimensions of the microstructure with the mode shapes provided in the Supplementary Material as the below ones: Young Modulus of *E* = 150GPa, the short beam elements of length *L*_1_ = 145.2 µm, the long beam element (supported beam) of length *L*_2_ = 221 µm, and the out-of-plane dimension of 25 µm. The beam and columns widths are respectively *h*_2_ = 1.84 µm and *h*_1_ = 1.8 µm. In addition, the distance between the supported beam and columns from their respective electrodes are *d* = 3.33 µm and *g* = 3.51 µm, respectively. These parameters are slightly different from the nominal values of the fabricated device (some with a 10% difference); however, they are within the range of measurement error and also correspond to Fig. 1. To yield simulated results, Eqs. (1) and (2) are integrated in time using the 4th order Runge–Kutta numerical method with the parameters of Table S1 calculated through the above-mentioned parameters.

### Experimental setup and characterization

**Figure 3:**
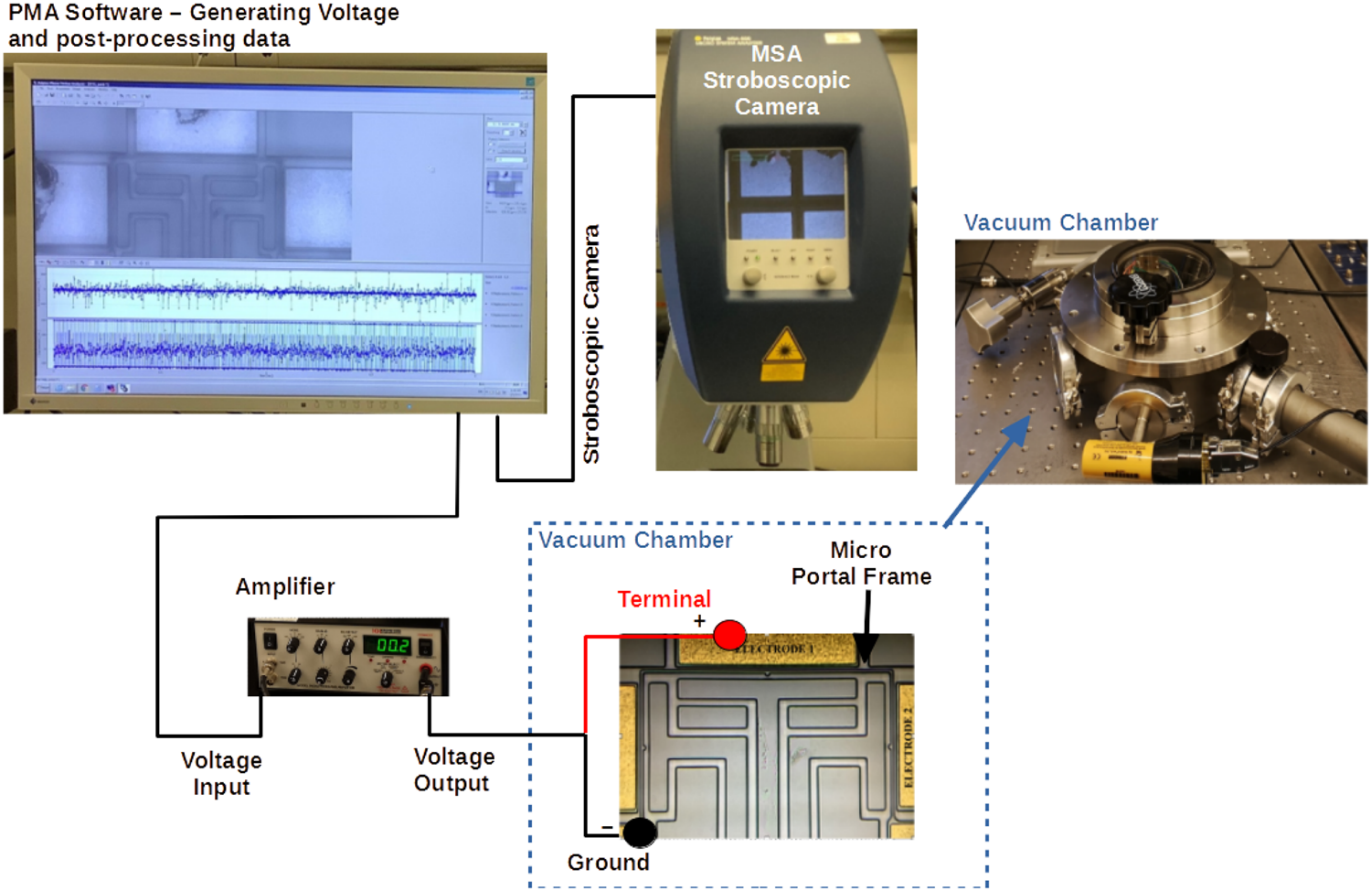
Experimental setup for the data acquisition of the micro portal frame.

The in-plane resonance frequencies are measured through the stroboscopic video microscopy of the Micro System Analyzer (MSA) from Polytec, Fig. 3, using a ring-down measurement. The camera’s targets are set on the end of a column, the square A, and in the mid-span of the supported beam, the square B, as illustrated in Fig. 1b, to measure the response of the 1st and 2nd modes, respectively. Square A is set to measure the displacement of the supported beam in the X-direction, while square B is set to measure the displacement of the structure in the Y-direction. Due to the small gap between the resonator and the electrodes, a vacuum chamber is required to minimize the squeeze-film damping^12^, with a pressure of 250mTorr set throughout the experiments.

The frequency responses curves, forced vibration responses, are obtained using the stroboscopic video microscopy with a Bode plot of the Planar Motion Analyzer (PMA) software of the MSA.

The first and second resonance frequencies are measured as 76.076 kHz and 166.710 kHz, respectively. The first mode is excited by actuating the portal frame with E1, while E2 actuates the second mode. Details of other higher-order modes are reported in^30^.

## Results

### Frequency tunability and 2:1 internal resonance condition

Originally, the device is designed to have a frequency ratio above 2:1 between the 1st and 2nd modes. Due to the electrostatic force, the resonance frequency of both modes can be tuned separately. Therefore, measurements and numerical simulations of the frequency shifts under the influence of the DC load through E2 (V_DC2_) are carried out. For calculating the natural frequency shift due to the electrostatic actuation, Eq. (S21) in the Supplementary Material is used.

**Figure 4:**
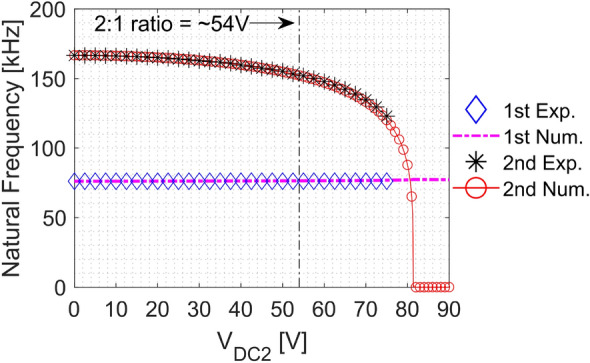
Natural frequency shifts of the first two modes due to the effect of the electrostatic force when actuating the structure through E2. Experimental data and numerical simulations show excellent agreement and reveal a possible 2:1 internal resonance at V_DC2_ = 54 V. Note that the actuation of the 2nd mode does not affect the 1st mode.

Figure 4 shows the frequency shift due to the DC actuation obtained throughout numerical simulations and experiments. Good agreement is noted between the model results and the experimental data. Both indicate that the natural frequency of the 2nd mode decreases with the increase of the DC load due to the softening effect of the electrostatic force. In addition, the 1st mode is not affected. Pull-in voltage is shown to be around V_DC2_ = 82 V. Around V_DC2_ = 54 V, a 2:1 ratio between the first and second modes is observed. The 2:1 ratio shows a potential internal resonance, which is the key condition for the saturation phenomenon.

### Nonlinear dynamic analysis

Next, through experiments, the structure is excited by an AC voltage through electrode E2 (V_AC2_) to investigate the dominant linear and nonlinear responses of the 2nd in-plane mode of the micro portal frame.

**Figure 5:**
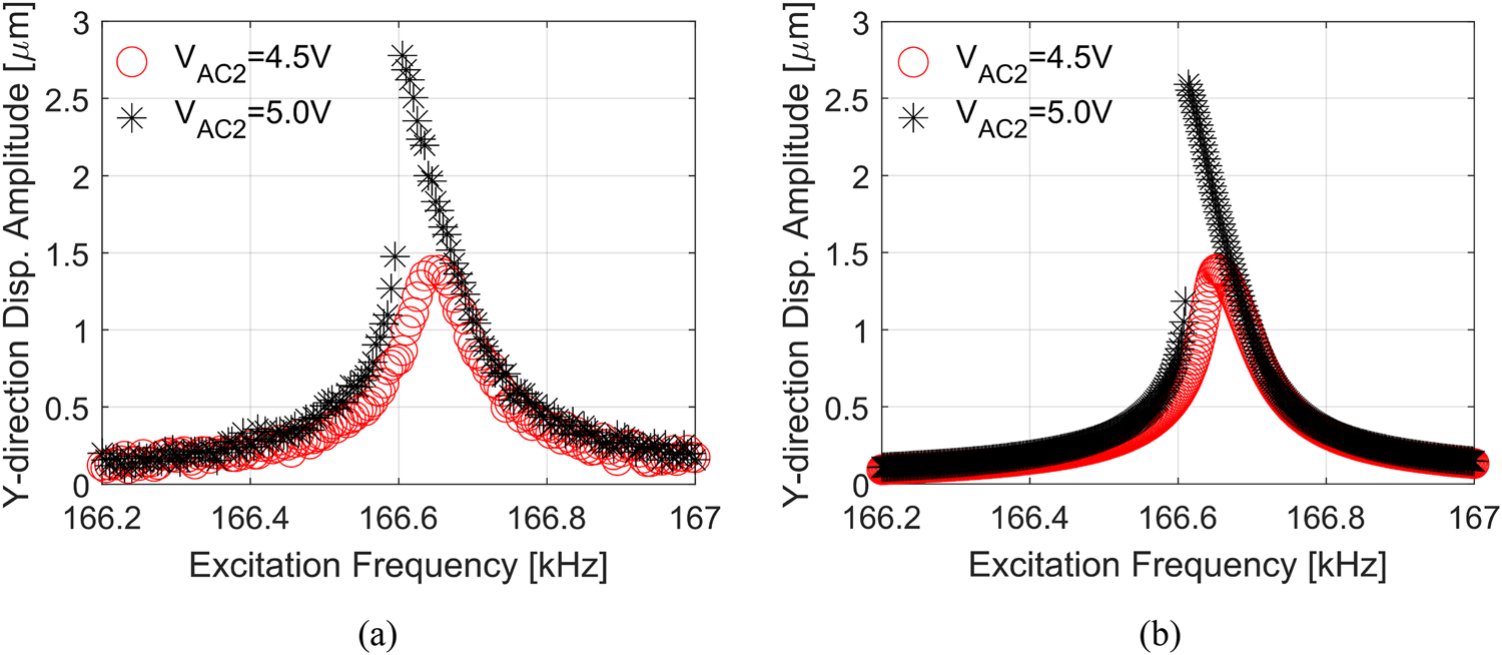
Frequency response curves near the 2nd resonance frequency with no DC load when actuating the structure with Electrode 2 (E2). (**a**) Experimental data. (**b**) Numerical simulations. Both figures indicate linear response and softening nonlinear behavior at V_AC2_ = 4.5 V and V_AC2_ = 5.0 V, respectively.

Figure 5a shows the frequency responses obtained through experiments. These results are obtained by analyzing the Y-direction motion of the middle point of the supported beam (square B in Fig. 1b). When the structure is subjected to V_AC2_ = 4.5 V without any DC load, linear response is observed. Increasing the electrostatic excitation to V_AC2_ = 5.0 V, nonlinear softening behavior dominates the response. Due to the absence of the DC load, the nonlinear response indicates the dominance of the geometric quadratic nonlinearities^30^. Figure 5b shows the frequency responses obtained by the numerical integration of Eqs. (1) and (2). Good agreement is noted between the experiments and numerical simulations.

As known for a straight clamped–clamped beam, hardening behavior is dominant due to mid-plane stretching. Softening nonlinear behavior prevails due to the effect of the increase of the DC load. However, as there is neither DC actuation nor internal resonance activation, in this case, the coefficient δ_2_ in Eq. (2) dominates the response. Although the nonlinear softening behavior has a qualitative agreement with the experimental data, it does not reach the same amplitude because the calculations slightly overestimate δ_2_. The coefficient should be around 80% of the current value for the amplitude agreement, although the slope increases. A complete parametric study of δ_2_ is presented in the Supplementary Material.

**Figure 6:**
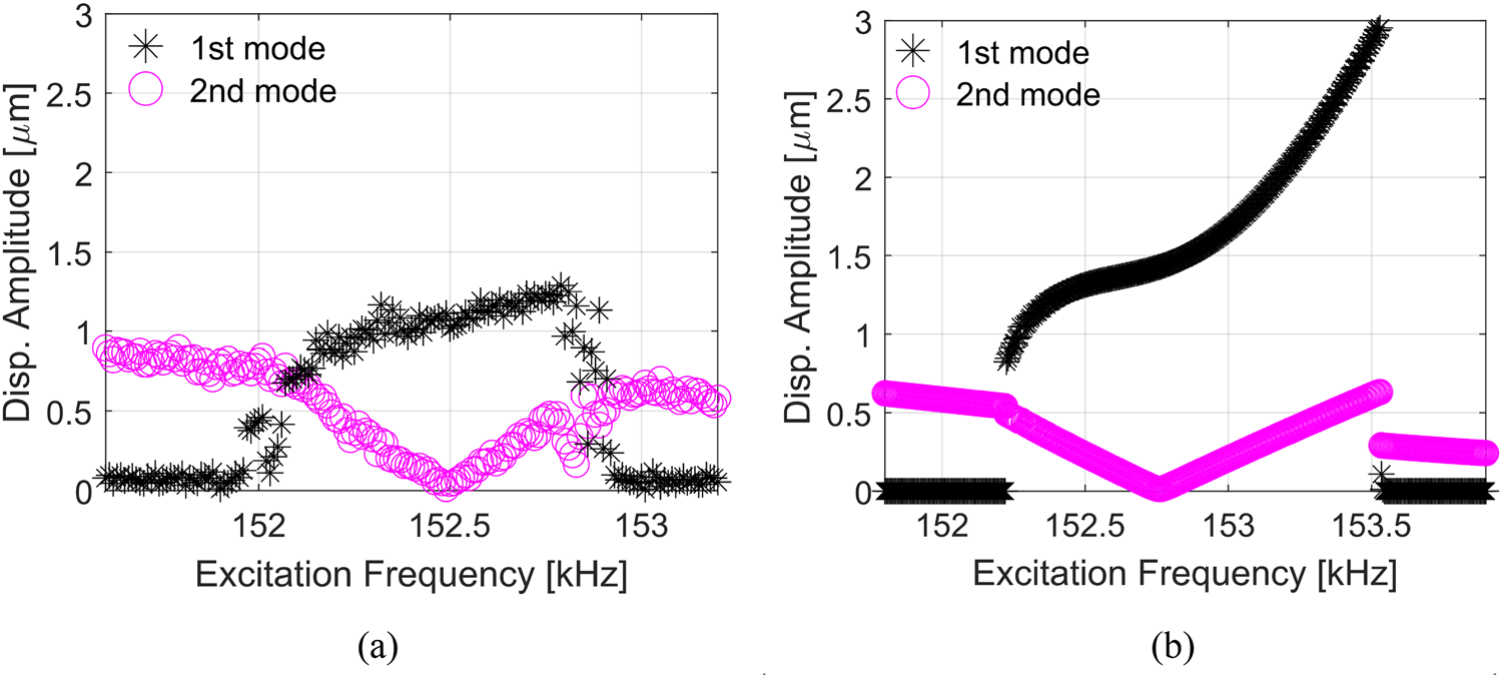
Frequency responses of the 1st and 2nd modes when exciting with E2 around the 2nd mode frequency. (**a**) Experimental data. (**b**) Numerical simulations. Note that due to the 2:1 internal resonance between the modes, the 1st mode, which was previously inactive, gets activated, while the response of the 2nd mode decreases. This shows that the surplus energy injected into the 2nd mode is transferred to the 1st mode.

At V_DC2_ = 54 V actuation with E2, Fig. 6a shows the experimental frequency response of the squares A and B of Fig. 1b, when exciting with V_AC2_ = 0.5 V around the 2nd resonance frequency. Note that around 152 kHz, the response of the 2nd mode decreases, while the 1st mode increases and becomes higher than the 2nd mode. Hence, the amplitude of the displacement of the columns is higher than the supported beam. This is one characteristic of the saturation phenomenon due to 2:1 internal resonance, as shown in^13–20,23^. In addition, Fig. 6b shows the numerical simulations under the same conditions as Fig. 6a, showing very good agreement with the experimental data. To illustrate the saturation phenomenon occurrence, an animation is shown in the supplementary Video 1. As a complement, the supplementary Video 2 shows the recorded motion of the portal frame after saturation phenomenon.

**Figure 7:**
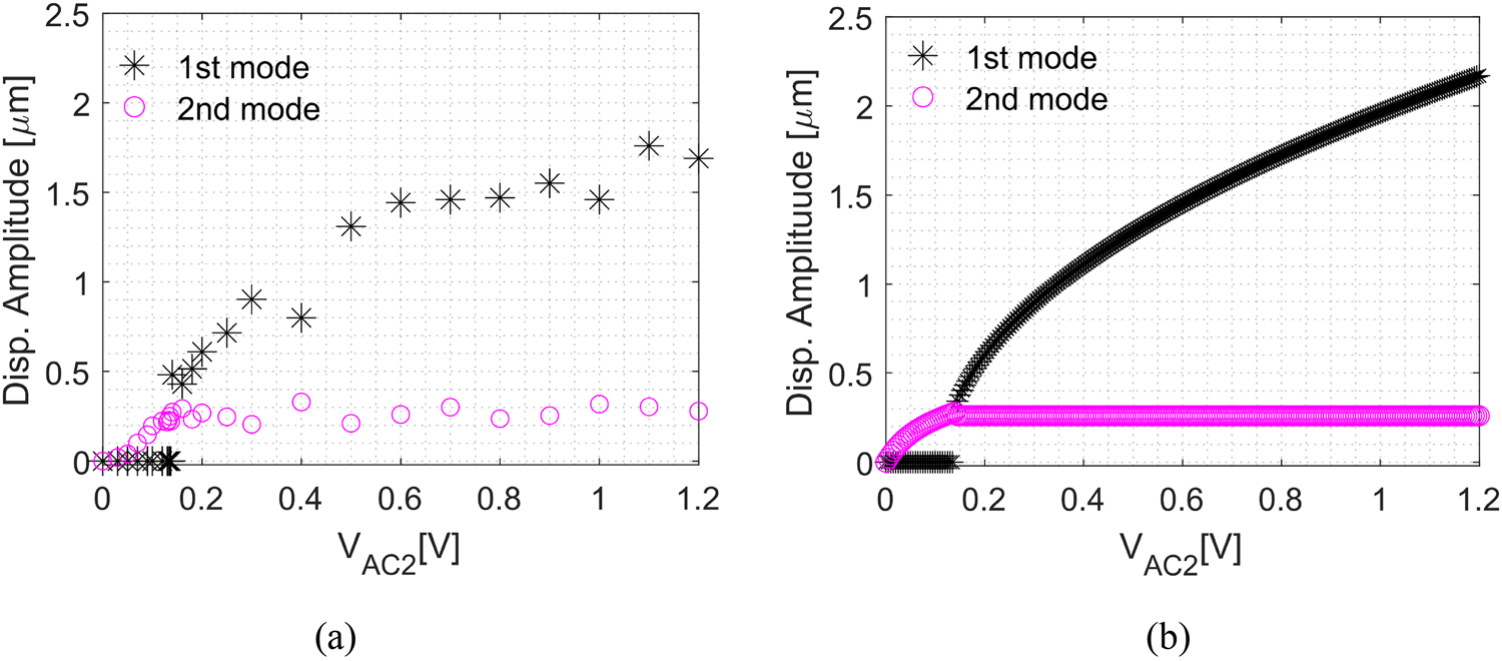
Force-response curves of the 1st and 2nd modes of the micro portal frame at V_DC2_ = 54 V actuation with E2. (**a**) Experimental data obtained at 152.3 kHz. (**b**) Numerical simulations at 152.47 kHz. The chosen frequencies of curves (**a**) and (**b**) are due to the equivalence of the response at Fig. 6. The force-response curves show the saturation of the 2nd mode around V_AC2_ = 140 mV, and all the extra vibration energy is transferred to the 1st mode.

To better demonstrate the saturation phenomenon, we refer to the force-response curve^13–15,18^. Figure 7 show the responses of the 1st and 2nd modes when varying the AC excitation obtained through experiments and numerical simulations. At the start, the amplitude of the directly excited 2nd mode increases with the increase of the AC excitation until V_AC2_ = 140 mV. At this specific voltage and critical amplitude, the response of the 2nd mode saturates (it does not change) with the increase of forcing/power. At the same time, the surplus energy is transferred to the 1st mode, which gets excited and its amplitude keeps increasing with the increase of the AC excitation^13^. The critical amplitude changes along with the resonance frequency within the 2:1 interval, which can be a valuable bifurcation point for potential bifurcation-based applications.

The most important condition for the saturation phenomenon occurrence is the 2:1 internal resonance. However, the occurrence of the phenomenon is not only limited to the exact 2:1 ratio. There is a parametrical domain, which changes the dominant nonlinear response after the phenomenon activation^32^, where the ratio between 1st and 2nd modes can be slightly higher or smaller than 2:1. In addition, the range of excitation frequencies that also activate the saturation may enlarge or shift.

**Figure 8:**
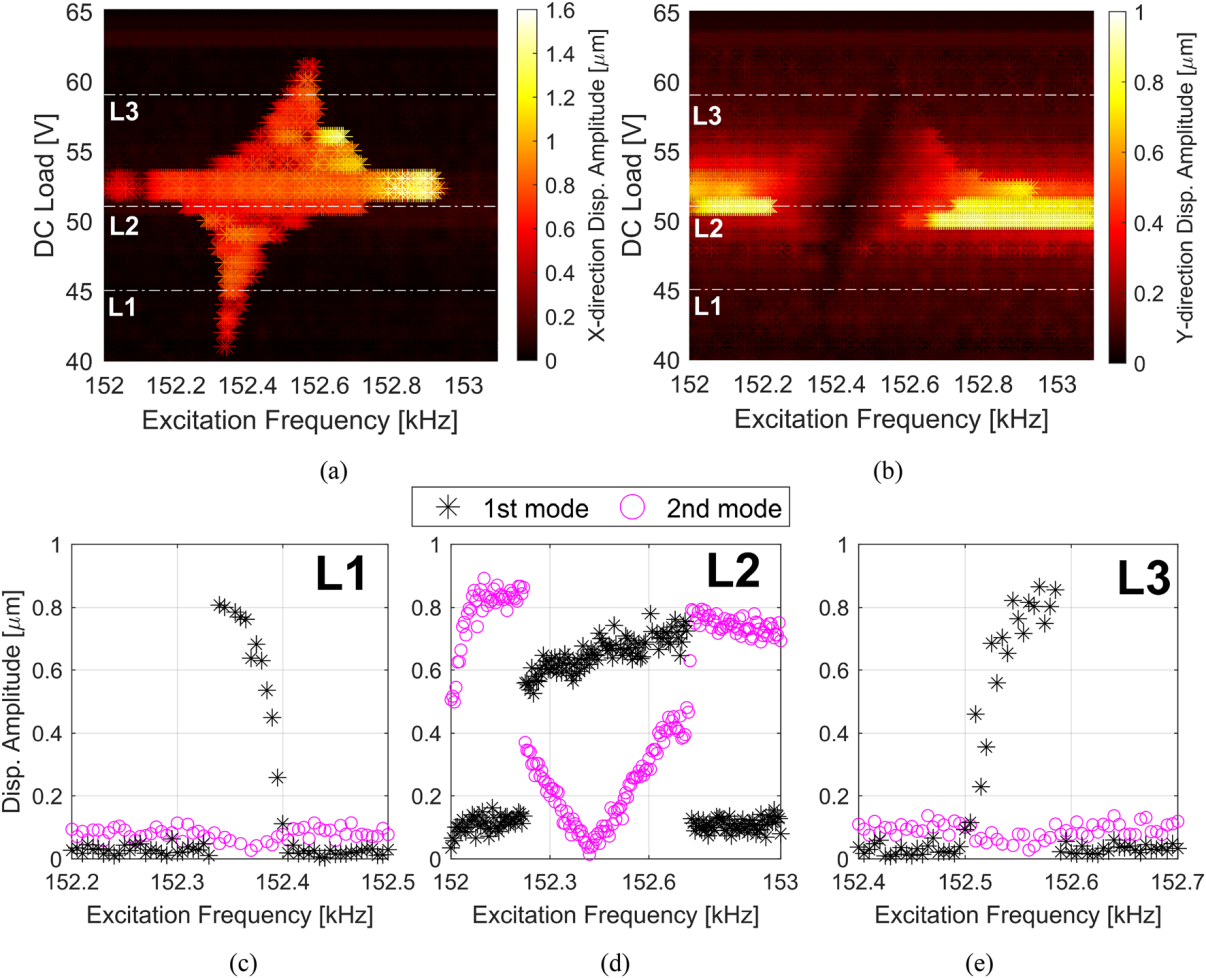
Experimental data of frequency responses surface (top view) for different DC loads through E2 excited around the 2nd mode frequency. (**a**) 1st mode. (**b**) 2nd mode. Both surfaces show the regions where the saturation phenomenon occurs, at which the 1st mode is activated. (**c**–**e**) Show the frequency responses of the 1st and 2nd modes for V_DC2_ = 45 V, V_DC2_ = 51 V, and V_DC2_ = 59 V, respectively. The curves show the different responses of the 1st mode under the saturation phenomenon when the ratios between 1st and 2nd modes are ω_2_/ω_1_ > 2, ω_2_/ω_1_ ≈ 2, and ω_2_/ω_1_ < 2, respectively. They also represent the dashed-white lines L1, L2, and L3 of the surfaces (**a**) and (**b**).

Figure 8a,b show the response of the 1st (X-direction displacement amplitude) and 2nd (Y-direction displacement amplitude) modes, respectively, throughout experiments, related to the DC load actuation through E2 and the range of frequencies around the 2:1 internal resonance. Note that within all the intervals where the saturation phenomenon occurs, the response of the 2nd mode is drastically reduced, while the 1st mode abruptly increases and can become higher than the 2nd mode.

In addition, as the DC load is tuned, it is expected that the resonance frequency of the 2nd mode may either increase or decrease. It can tune the ratio between the 2nd resonance frequency and the 1st one higher or lower than ω_2_ = 2ω_1_. Depending on this ratio, the response of the 1st mode due to the saturation phenomenon changes, turning out in different bifurcation points and frequency intervals of interest. To show that, three (3) frequency response curves denoted by the dashed lines L1, L2, and L3 are highlighted and discussed, where L1, L2, and L3 use different DC actuation, yielding the ω_2_/ω_1_ ratios according to Table 1.

**Table 1:** 1st and 2nd modes of vibration ratios close to the 2:1.

Line	DC Load through E2 [V]	ω_2_/ω_1_
L1	45	2.067 (> 2)
L2	51	2.021 (≈ 2)
L3	59	1.938 (< 2)

Figure 8c–e show the frequency responses of the 1st and 2nd modes of L1, L2, and L3 lines highlighted in Fig. 8a,b. In line L1, where the ratio is higher than ω_2_/ω_1_ > 2, the saturation phenomenon occurs when the structure is excited around twice ω_1_, and the nonlinear softening behavior is observed from the outcome of the 1st mode. The phenomenon is also realized when the ratio is smaller than 2:1, as shown by the frequency response of line L3. On the other hand, hardening behavior is noted from the response of the 1st mode. Although the resonance frequencies of the 2nd mode at lines L1 (ω_2_ = 157.6 kHz) and L3 (ω_2_ = 148.1 kHz) are far from the exact 2:1 ratio (ω_2_ = 154.3 kHz), the saturation phenomenon is still achievable.

When the ratio is very close to 2:1, the classical “V-shape” curve of the saturation phenomenon is observed either for the 1st or 2nd mode, as shown in the response of line L2. In addition, it is important to highlight that the saturation phenomenon dominates the response over any other nonlinearity. This is observed in Figs. 6 and 8d, as the phenomenon is presented dominant over the nonlinear softening behavior induced by the electrostatic force.

For a more comprehensive overview, Fig. S3 in the Supplementary Material shows the numerically simulated responses of Fig. 8. In addition, Fig. S3b highlights the shift of the nonlinear softening behavior along with the tune of the DC load. The numerical simulations show a qualitative agreement with the experimental results, demonstrating that the mathematical model can support further researches on the MEMS/NEMS U-shape portal frame.

## Discussion

The in-plane micro-electromechanical portal frame (U-shape) structure has the advantage of being of multi-input and multi-output device, which can be utilized for applications in sensing, signal filtering, logic, and energy harvesting^7,28,29^. Its multi-channel feature can provide various tunability options with different ports/electrodes.

In addition, the output actuation can be amplified through internal resonance and can be in the direction orthogonal to the input excitation (for example, inputting through the first sway mode and outputting through the bending second mode). Hence, achieving a precise ratio for internal resonance and saturation, such as 2:1, can be easily achieved despite fabrication imperfections. For instance, if the fabricated portal frame has ω_2_/ω_1_ < 2, tunability is provided by actuating the columns so that ω_1_ decreases. In this work, the portal frame was fabricated with ω_2_/ω_1_ > 2. Hence, actuating it through the supporting middle beam decreased the ratio to the desired 2:1 ratio.

This design shows high potential for applications in bifurcation-based sensors^33^, where one can excite the system outside the internal resonance band, and upon the detection of a physical quantity like mass, the inactive mode gets activated, and a high signal can be obtained as a clear indication of sensing. This is possible by observing Fig. 8 as there is an operational frequency band where the saturation phenomenon occurs and is possible to be achieved due to the frequency tunability.

For instance, a magnetic-based sensor can be used to tune the resonance frequency^34^. Depending on the magnetic field strength, the operational frequency can be driven to the activation of the internal resonance. This is in addition to the potential for using the device for frequency stabilization and lowering noise (noise at the output is absorbed in the input, leading to high signal–noise ratio at the output).

## Supplementary Information

Supplementary Information 1.

Supplementary Video 1.

Supplementary Video 2.
