# Supplementary material for: Nonlinear mode saturation in a U-shaped micro-resonator
Source: Sci Rep. 2022 Jun 21;12:10420. doi: 10.1038/s41598-022-14657-1 (PMC9213437; doi:10.1038/s41598-022-14657-1)
Supplement: Supplementary file 1 — Supplementary Information 1. [file 41598_2022_14657_MOESM1_ESM.docx]

## Nonlinear Mode Saturation in a U-Shaped Micro-Resonator

## Supplementary Material: Mathematical Modelling

Rodrigo T. Rocha and Mohammad I. Younis

Physical Sciences and Engineering Division, King Abdullah University of Science and Technology – KAUST, Thuwal 23955, Saudi Arabia

A mathematical model is developed to simulate the dynamic response of the system. For this, the micro portal frame is represented by Fig. S1. The supported middle beam is represented by the lumped mass *m_b_* and an area moment of inertia *I_b_*. A lumped mass *m_c_* represents each vertical column with an area moment of inertia *I_c_*. Both columns and beam have a depth *b*. In this reduced-order mathematical model, inextensible links are considered. Geometric parameters of the structure are given in the subsection Fabricated Device of the main manuscript.


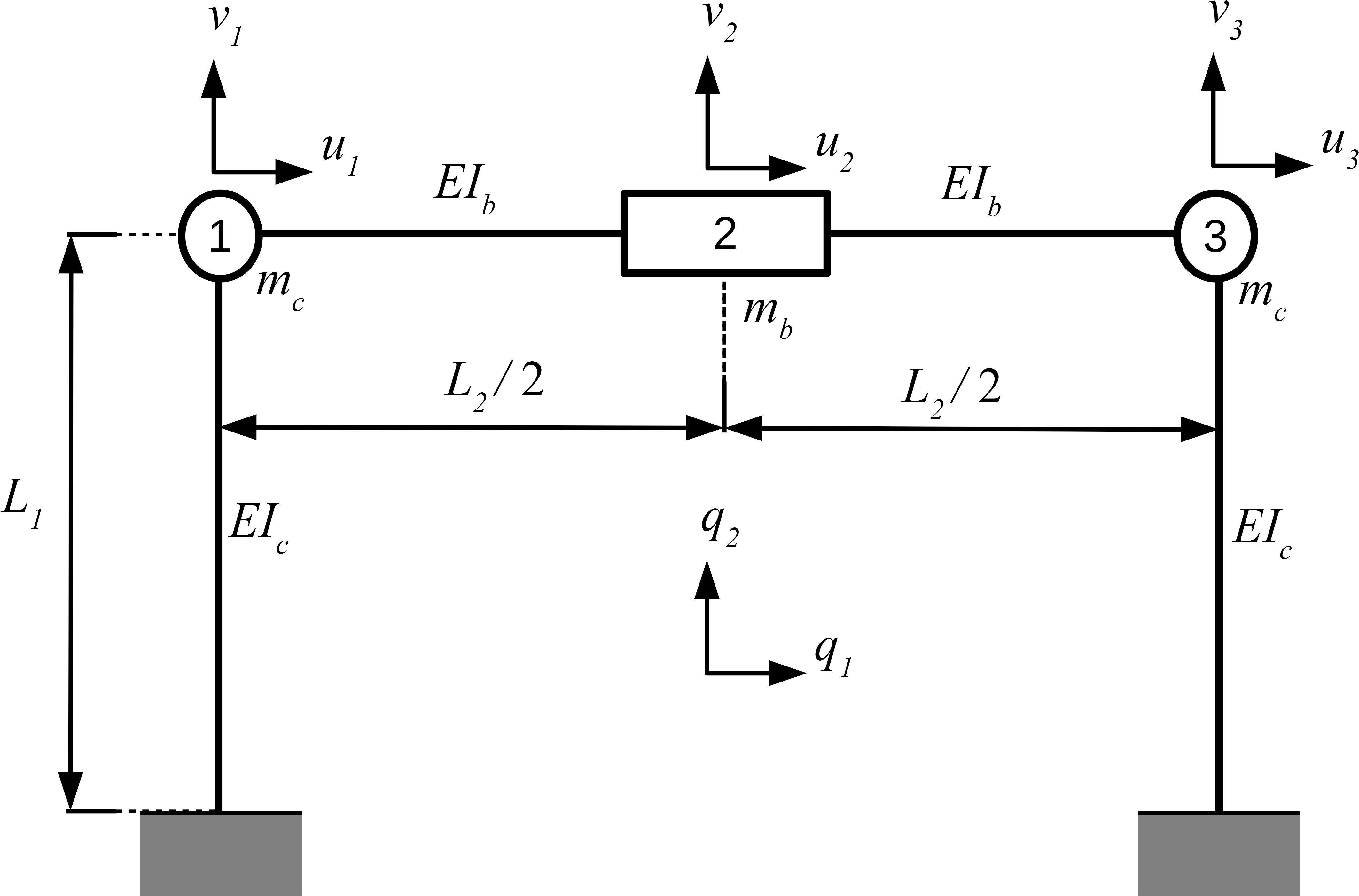


Figure S1 – Schematic of the micro portal frame model.

The linear stiffness of the columns and the beam can be evaluated by a finite-element Rayleigh-Ritz procedure [S1]. The shortening due to bending of the columns and beam introduces quadratic geometric nonlinearity of the structure. The shape function ­ related to the columns and beam are obtained by solving Eq. (S37) admitting , which yields

(S1)

where $\gamma=\frac{L_{2}}{L_{1}}\sqrt{\frac{h_{1}}{h_{2}}}$, *A­_1,3_-D_1,3_* are unknown constants, the index *j* is the mode of vibration, *ω_non_* is the non-dimensional frequency, and $\hat{x}$*,* $\hat{y}$, and $\hat{z}$ are the non-dimensional length of each beam element.

The structure is subjected to the boundary conditions obtained through Hamilton’s principle assuming symmetry developed in the Mode Shape Developments section, which are expressed in the non-dimensional form as

(S2)

Hence, using Eq. (S2) along with Eqs. (S1), the mode shapes for the 1^st^ and 2^nd^ modes are written as

(S3a)

(S3b)

(S3c)

and

(S3d)

(S3e)

(S3f)

where and refer to the mode shapes of the free vibration of the 1^st^ and 2^nd^ modes, respectively, and are shown in Fig. S2. As both modes account for the X- and Y- direction motions of the mid-span of the supported beam, further calculations use only Eqs. (S3a) and (S3e) for the respective modes.


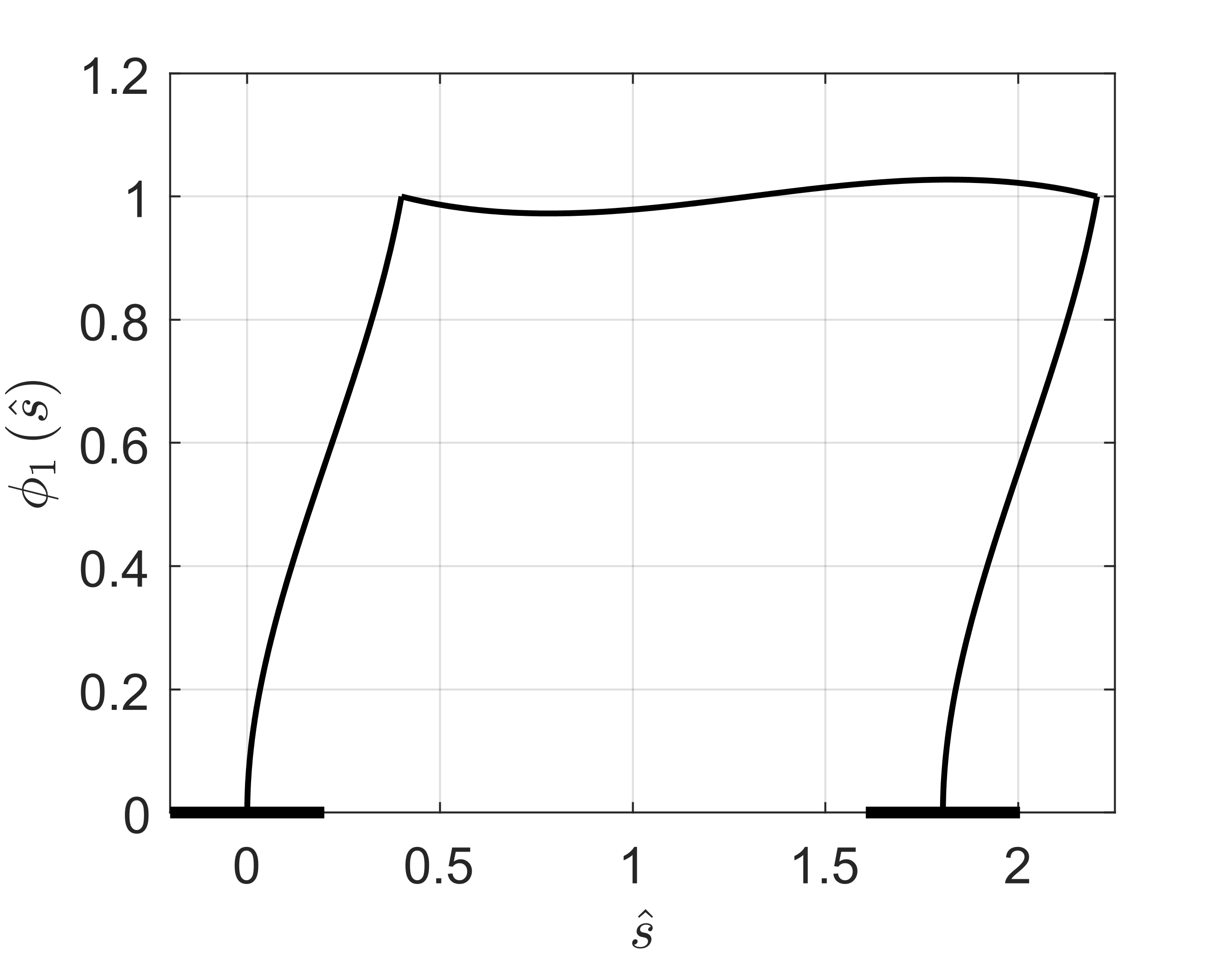

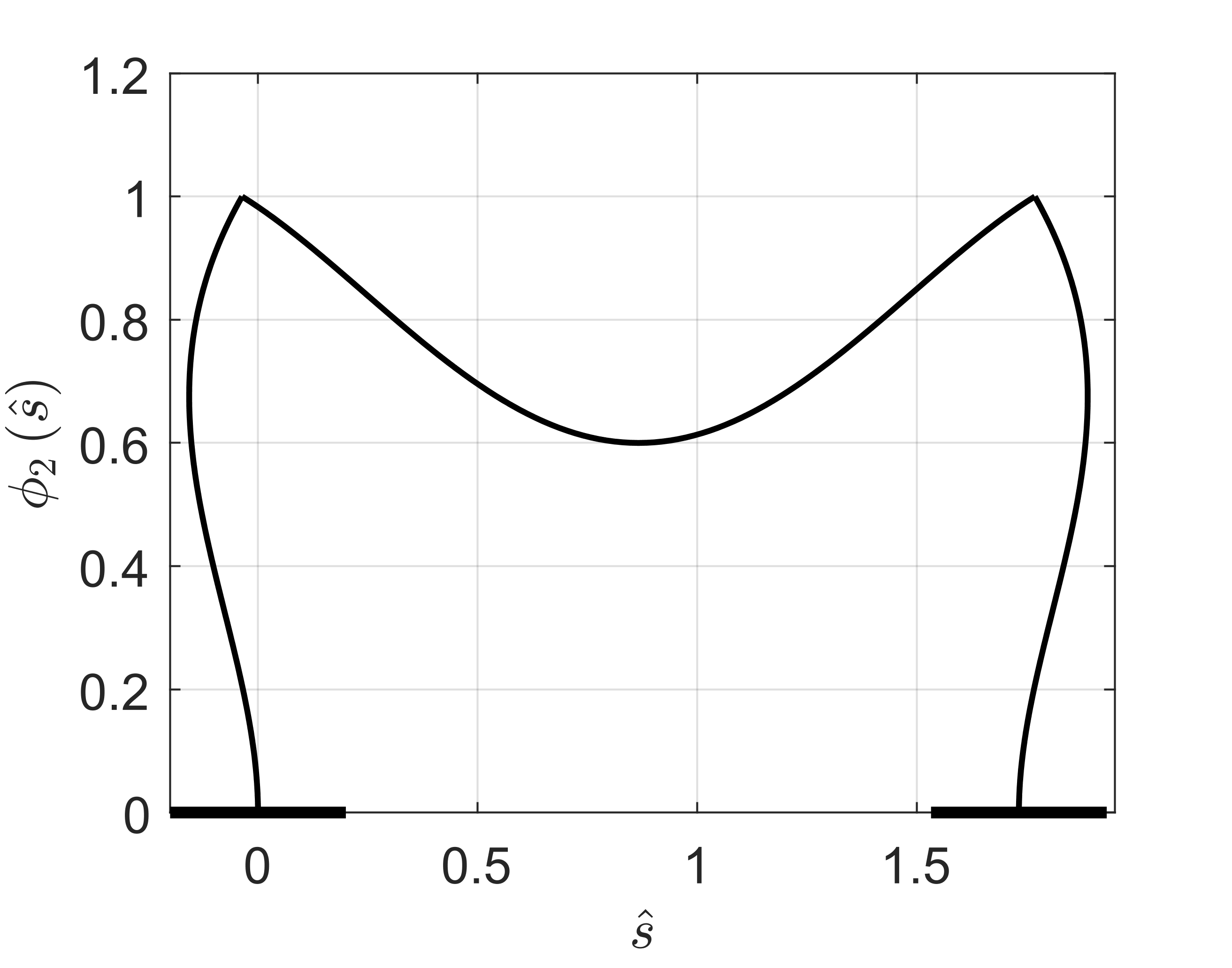


(a) (b)

Figure S2 – Mode shape curves of the (a) 1^st^ mode (from Eqs. (S3a)- (S3c)), and the (b) 2^nd^ mode (from Eqs. (S3d)- (S3f)). The mode shape of the 1^st^ mode is normalized by 2.5 times the maximum value of the column mode shape, computed as  and the 2^nd^ mode shape is normalized by 2.5 times the maximum value of the supported beam mode shape, computed as . In addition, the lengths of the mode shapes are adjusted to have the ratio between *L_2_*/*L_1_* for maintaining the scale.

The nodal displacements, shown in Fig. S1, are related to the generalized coordinates *q_1_* and *q_2_* of the structure, which are set at the mid-span of the supported beam, as

$\begin{matrix} u_{2}=q_{1}\text{ }u_{1}=u_{2}+\frac{B}{2}v_{2}^{2}\text{ }u_{3}=u_{2}-\frac{B}{2}v_{2}^{2} \\ v_{2}=q_{2}\text{ }v_{1}=-\frac{C}{2}u_{2}^{2}\text{ }v_{3}=-\frac{C}{2}u_{2}^{2} \end{matrix}$ (S4)

where the constants *C* and *B* are as below

(S5a)

We calculate the linear stiffness of the beam *k_b_* and columns *k_c_* through Rayleigh’s method as

(S5b)

The total kinetic energy of the structure *T* is calculated by accounting for the motion of all the three masses related to the nodal displacement as

(S6)

where the masses are calculated per unit length as

(S7)

where *ρ* is the mass density of the material. Hence, deriving Eq. (S4) in time and substituting it into Eq. (S6) yields

(S8)

The potential energy of the structure *U* is given by

$U=\frac{1}{2}k_{c}\left(u_{1}^{2}+u_{3}^{2}\right)+\frac{1}{2}k_{b}\left(v_{2}-\frac{v_{1}+v_{3}}{2}\right)^{2}$ (S9)

Substituting the nodal displacements of Eq. (S4) into Eq. (S9) yields

$U=k_{c}q_{2}^{2}+\frac{1}{2}Ck_{b}q_{1}q_{2}^{2}+\frac{1}{2}k_{b}q_{1}^{2}+\frac{1}{4}C^{2}k_{b}q_{2}^{4}+\frac{1}{8}B^{2}k_{c}q_{1}^{4}$ (S10)

The potential energy shows quadratic, cubic, quartic, and coupling terms. The dissipative energy *D* of the supported beam and columns is defined as

(S11)

where *c_c_* and *c_b_* are the damping coefficients of the columns and supported beam, respectively.

The structure is actuated by DC and AC electrostatic loads through Electrode 1 and Electrode 2. Hence, the electrostatic force for each electrode is given by

$\begin{matrix} F_{\text{E}1}=\frac{\varepsilon A_{1}}{2\left(g-u_{1}\right)^{2}}\left(V_{DC1}+V_{AC1}\cos\omega_{1}t\right)^{2} \\ F_{\text{E2}}=\frac{\varepsilon A_{2}}{2\left(d-v_{2}\right)^{2}}\left(V_{DC2}+V_{AC2}\cos\omega_{2}t\right)^{2} \end{matrix}$ (S12)

where ε is the dielectric constant, *A_1_* and *A_2_* are the actuation area of each electrode 1 and 2, and ω_1_ and ω_2_ are the excitation frequency of electrode 1 and 2, respectively. Note that the electrostatic forces are related to the nodal displacements through Eq. (S4), and that the quadratic term of *u_1_* from Eq. (S4) is neglected for F_E1_ in Eq. (S12), as its influence is negligible for the results.

Next, we apply the Euler-Lagrange equations

(S13)

(S14)

where *Q_ext_* is the generalized non-conservative forces.

Hence, the equations of motion of the portal frame are obtained as

(S15)

where the first and second equations represent the X-direction (1^st^ mode) and Y-direction (2^nd^ mode) displacements, respectively.

A dimensionless procedure is carried out with the new non-dimensional variables below

$x=\frac{q_{1}}{g}\text{; }y=\frac{q_{2}}{d}\text{; }\omega_{n1}=\sqrt{\frac{2k_{c}}{2m_{c}+m_{b}}}\text{; }\tau=\omega_{n1}t;\text{ }c_{c}=2m_{c}\omega_{n1}\zeta_{c};\text{ }c_{b}=2m_{b}\omega_{n1}\zeta_{b};$ (S16)

yielding the new non-dimensional equations of motion

(S17)

where the dimensionless coefficients are

$\begin{matrix} k_{31}=\frac{C^{2}g^{2}k_{b}}{4k_{c}}\text{; }\alpha_{1}=\frac{Cdk_{b}}{2k_{c}};\text{ }\delta_{1}=\frac{2C^{2}g^{2}m_{c}}{\left(m_{b}+2m_{c}\right)}\text{; } \text{ }\mu_{1}=\frac{2m_{c}\zeta_{c}}{\left(m_{b}+2m_{c}\right)}; \\ \Omega_{1}=\frac{\omega_{1}}{\omega_{n1}};\text{ }\beta_{1}=\frac{\varepsilon A_{1}}{4g^{3}k_{c}}\text{; }\beta_{2}=\frac{\varepsilon A_{2}\left(m_{b}+2m_{c}\right)}{4d^{3}k_{c}m_{b}}; \text{ }k_{32}=\frac{B^{2}d^{2}\left(m_{b}+2m_{c}\right)}{2m_{b}}\text{; } \\ \omega_{n2}\text{=}\sqrt{\frac{\left(m_{b}+2m_{c}\right)k_{b}}{2k_{c}m_{b}}}\text{; }\alpha_{2}=\frac{Cg^{2}k_{b}\left(m_{b}+2m_{c}\right)}{4dk_{c}m_{b}};\text{ }\delta_{2}=\frac{2B^{2}d^{2}m_{c}}{m_{b}}\text{; } \text{ }\mu_{2}=2\zeta_{b};\text{ }\Omega_{2}=\frac{\omega_{2}}{\omega_{n1}}\text{;} \end{matrix}$(S18)

and with their values given in Table S1.

Table S1 – Parameters’ values of Eq. (S18).

| Parameter | Value |
| --- | --- |
| *k*_31_ | 1.76×10^-3^ |
| *k*_32_ | 4.19×10^-3^ |
| *α*_1_ | 0.101 |
| *α*_2_ | 0.084 |
| *β*_1_ | 2.22×10^-5^ |
| *β*_2_ | 1.06×10^-4^ |
| *δ*_1_ | 3.48×10^-4^ |
| *δ*_2_ | 2.71×10^-3^ |
| *µ*_1_ | 1.84×10^-4^ |
| *µ*_2_ | 1.14×10^-3^ |
| *ω*_n2_ | 2.19 |

# Static Deflection and Eigenvalue Matrices

For calculating the natural frequency shift due to the DC and AC electrostatic actuation, static deflections *x*_s_ and *y*_s_ are assumed in Eq. (S17). Hence, dropping the time-dependent terms yields

$\begin{matrix} x_{s}+k_{31}x_{s}^{3}+\alpha_{1}x_{s}y_{s}=\beta_{1}\frac{\left(V_{AC1}^{2}+2V_{DC1}^{2}\right)}{2\left(1-x_{s}\right)^{2}} \\ \omega_{n2}^{2}y_{s}+k_{32}y_{s}^{3}+\alpha_{2}x_{s}^{2}=\beta_{2}\frac{\left(V_{AC2}^{2}+2V_{DC2}^{2}\right)}{2\left(1-y_{s}\right)^{2}} \end{matrix}$ (S19)

Next, we perturb Eq. (S17) around the equilibrium state by assuming the states composed of the static and dynamic parts *x_d_* and *y_d_*, *x* = *x_d_* + *x_s_* and *y* = *y_d_* + *y_s_*, and extract the mass **M** and stiffness **K** matrices dependent on *x_d_* and *y_d_* as

$\begin{matrix} \text{ }M=\left[\begin{matrix} 1+\delta_{1}x_{s}^{2} & 0 \\ 0 & 1+\delta_{2}y_{s}^{2} \end{matrix}\right]; \\ K=\left[\begin{matrix} 1+3k_{31}x_{s}^{2}+\alpha_{1}y_{s}-\frac{\beta_{1}\left(V_{AC1}^{2}+2V_{DC1}^{2}\right)}{\left(1-x_{s}\right)^{3}} & \alpha_{1}x_{s} \\ 2\alpha_{2}x_{s} & \omega_{n2}^{2}+3k_{32}y_{s}^{2}-\frac{\beta_{2}\left(V_{AC2}^{2}+2V_{DC2}^{2}\right)}{\left(1-y_{s}\right)^{3}} \end{matrix}\right]; \end{matrix}$ (S20)

Hence, the eigenvalue problem is solved according to

$\left(K-\lambda M\right)q=0$ (S21)

where λ_k_(ω_n1_/2π) yields the natural frequencies of the first (*k* = 1) and second (*k* = 2) modes of the system, λ is the eigenvalue, and *q* is the eigenvector.

# Saturation Phenomenon Analysis for DC load vs Excitation Frequency

Figures S3a and S3b show the simulated response of the 1^st^ and 2^nd^ modes, respectively, related to the DC load actuation through E2 and the range of frequencies around the 2:1 internal resonance. Note that within all the intervals where the saturation phenomenon occurs, the response of the 2^nd^ mode is drastically reduced. In contrast, the 1^st^ mode abruptly increases and can become higher than the 2^nd^ mode.

In addition, as the DC load is tuned, it is expected that the resonance frequency of the 2^nd^ mode may either increase or decrease. It can tune the ratio between the 2^nd^ resonance frequency and the 1^st^ one higher or lower than ω­_2_ = 2ω­_1_. Depending on this ratio, the response of the 1^st^ mode due to the saturation phenomenon changes, turning out in different bifurcation points and frequency intervals of interest. To show that, three (3) frequency response curves denoted by the dashed lines L1, L2, and L3 are highlighted and discussed, where L1, L2, and L3 use different DC actuation, yielding the ω­_2_/ω­_1_ ratios according to Table S2.

Table S2 – 1^st^ and 2^nd^ modes of vibration ratios close to the 2:1.

| Line | DC Load through E2 [V] | ω­_2_/ω­_1_ |
| --- | --- | --- |
| L1 | 45 | 2.064 (> 2) |
| L2 | 53.2 | 2 |
| L3 | 65 | 1.847 (< 2) |

Figs. S3c, S3d, and S3e show the frequency responses of the 1^st^ and 2^nd^ modes of L1, L2, and L3 lines highlighted in Figs. S3a, and S3b. In line L1, where the ratio is higher than ω­_2_/ω­_1_ > 2, the saturation phenomenon occurs when the structure is excited around twice ω­_1_, and the nonlinear softening behavior is observed from the outcome of the 1^st^ mode. The phenomenon is also realized when the ratio is smaller than 2:1, as shown by the frequency response of line L3. On the other hand, hardening behavior is noted from the response of the 1^st^ mode. Although the resonance frequencies of the 2^nd^ mode at lines L1 (ω­_2_ = 157.6 kHz) and L3 (ω­_2_ = 141.4 kHz) are far from the exact 2:1 ratio (ω­_2_ = 152.8 kHz), the saturation phenomenon is still achievable.

When the ratio is exactly 2:1, the classical “V-shape” curve of the saturation phenomenon is observed either for the 1^st^ or 2^nd^ modes, as shown in the response of line L2. In addition, it is important to highlight that the saturation phenomenon dominates the response over any other nonlinearity. This is observed in Figs. 6, 8d, and S3d, as the phenomenon is presented dominant over the nonlinear softening behavior induced by the electrostatic force.


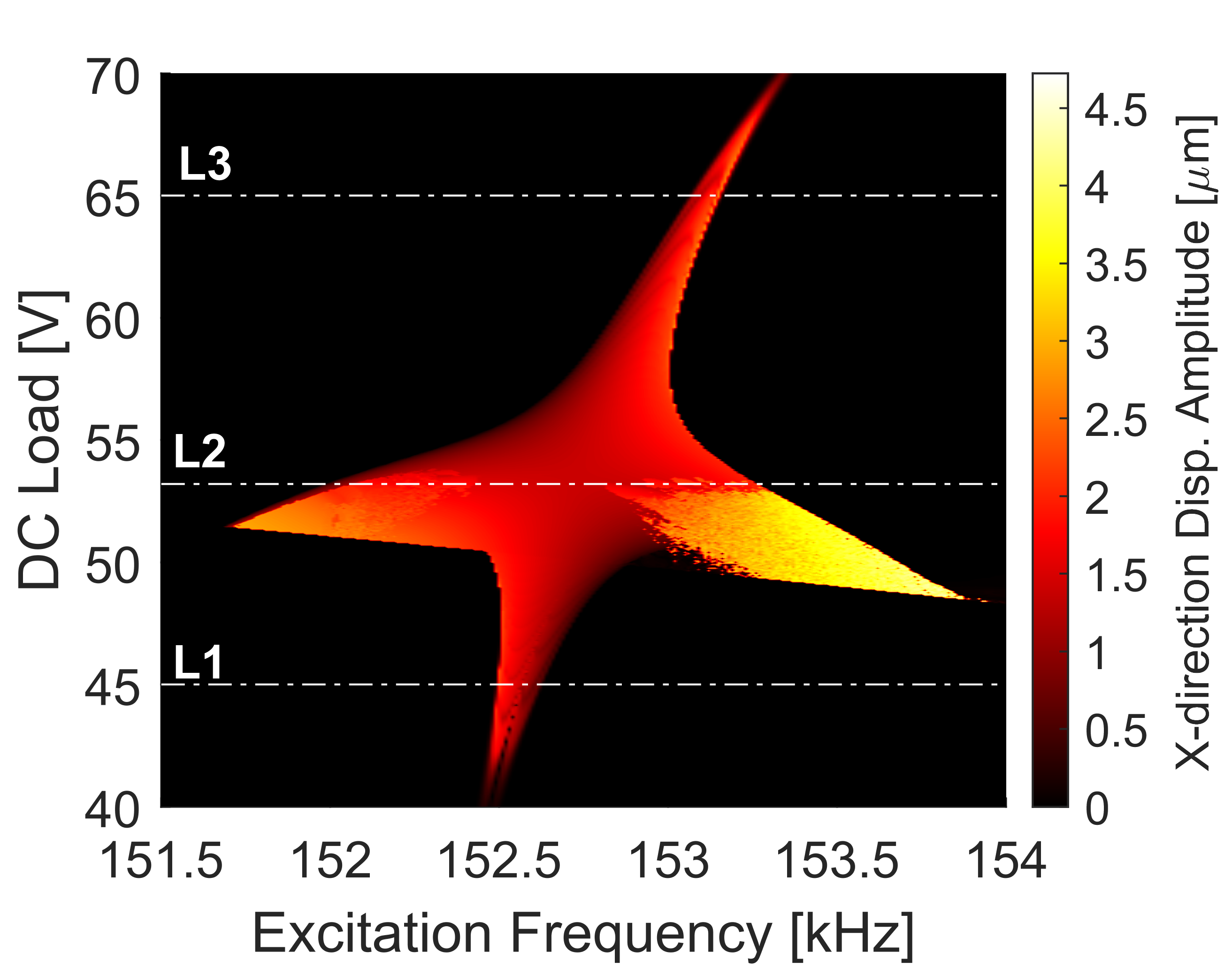

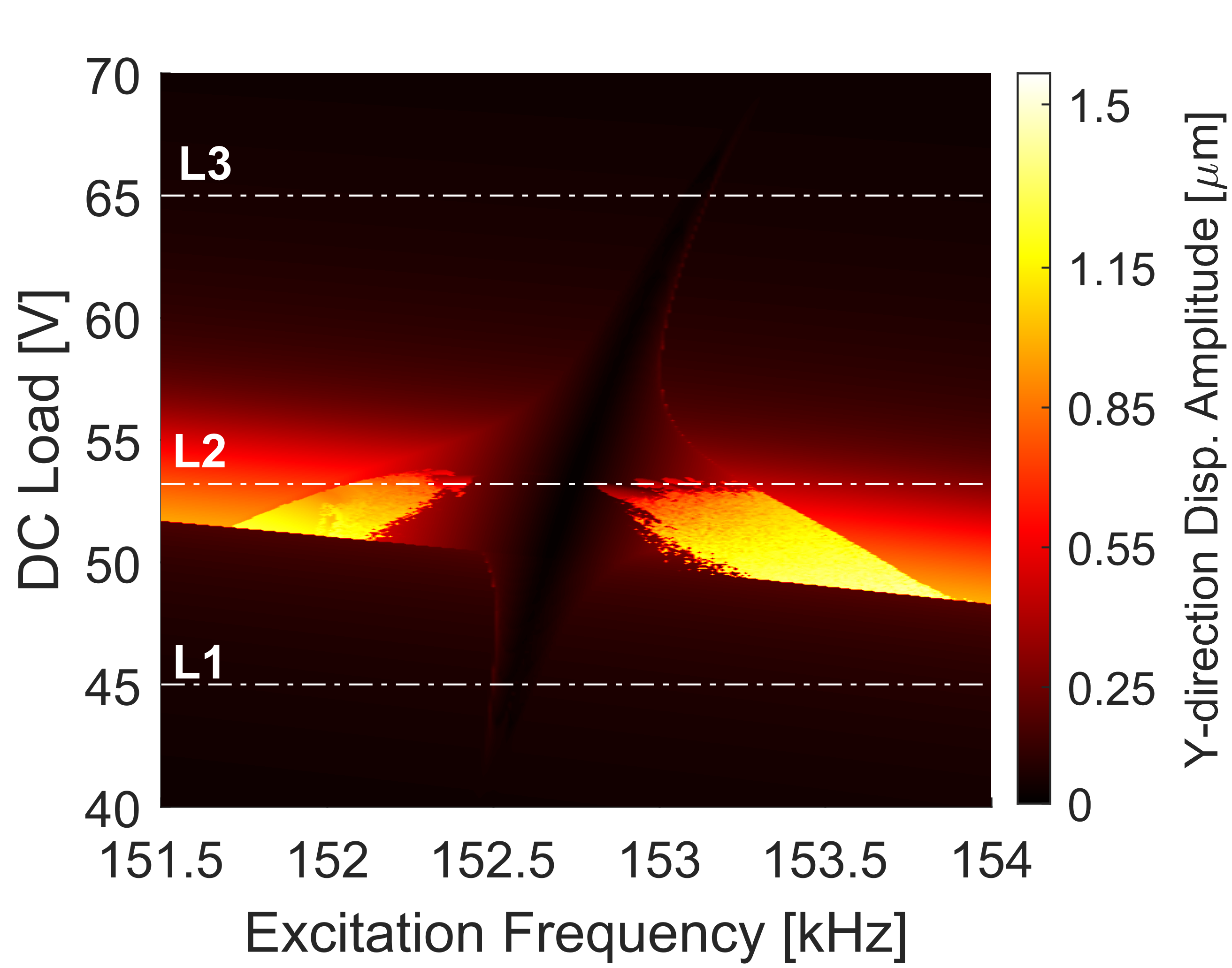


2s

1. (b)


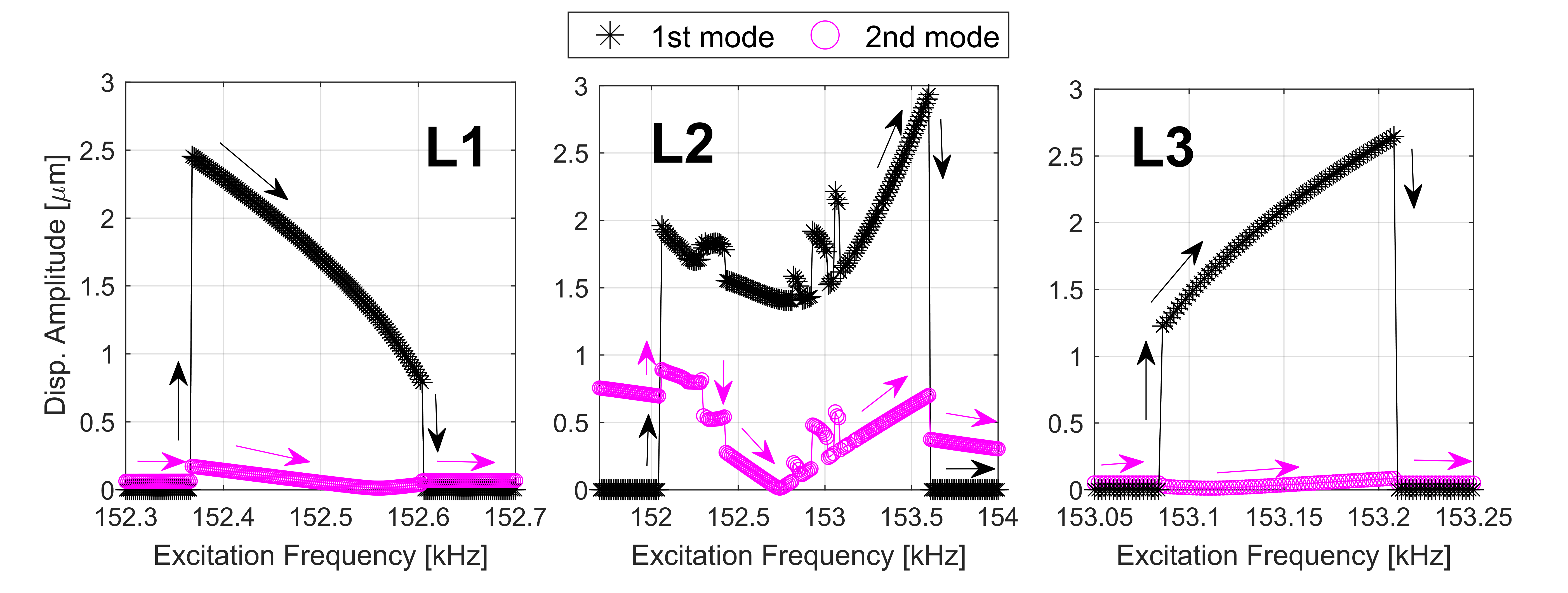


(c) (d) (e)

Figure S3 – Numerical simulations of frequency responses surface (top view) for different DC loads through E2 excited around the 2^nd^ mode frequency. (a) 1^st^ mode. (b) 2^nd^ mode. Both surfaces show the regions where the saturation phenomenon occurs, at which the 1^st^ mode is activated. (c), (d), and (e) show the frequency responses of the 1^st^ and 2^nd^ modes for V_DC2_ = 45V, V_DC2_ = 53.2V, and V_DC2_ = 65V, respectively. The curves show the different responses of the 1^st^ mode under the saturation phenomenon when the ratios between 1^st^ and 2^nd^ modes are ω­_2_/ω­_1_>2, ω­_2_/ω­_1_ = 2, and ω­_2_/ω­_1_<2, respectively. They also represent the dashed-white lines L1, L2, and L3 of the surfaces (a) and (b).

# Pressure vs Saturation Phenomenon

The saturation phenomenon is also known to be influenced by damping. For MEMS devices, damping can be controlled by tuning the pressure inside an environmental chamber. At V_DC2_ = 54V, Figs. S4a and S4b show experimental frequency responses of the 1^st^ and 2^nd^ modes when excited with V_AC2_ = 0.5V around the 2^nd^ resonance frequency for different pressure values. Note that the saturation phenomenon occurs for high pressures around 2Torr, which is enough even to reduce the nonlinear softening behavior induced by the electrostatic force. It is also observed that the higher the pressure, the shorter is the frequency bandwidth that the phenomenon occurs.


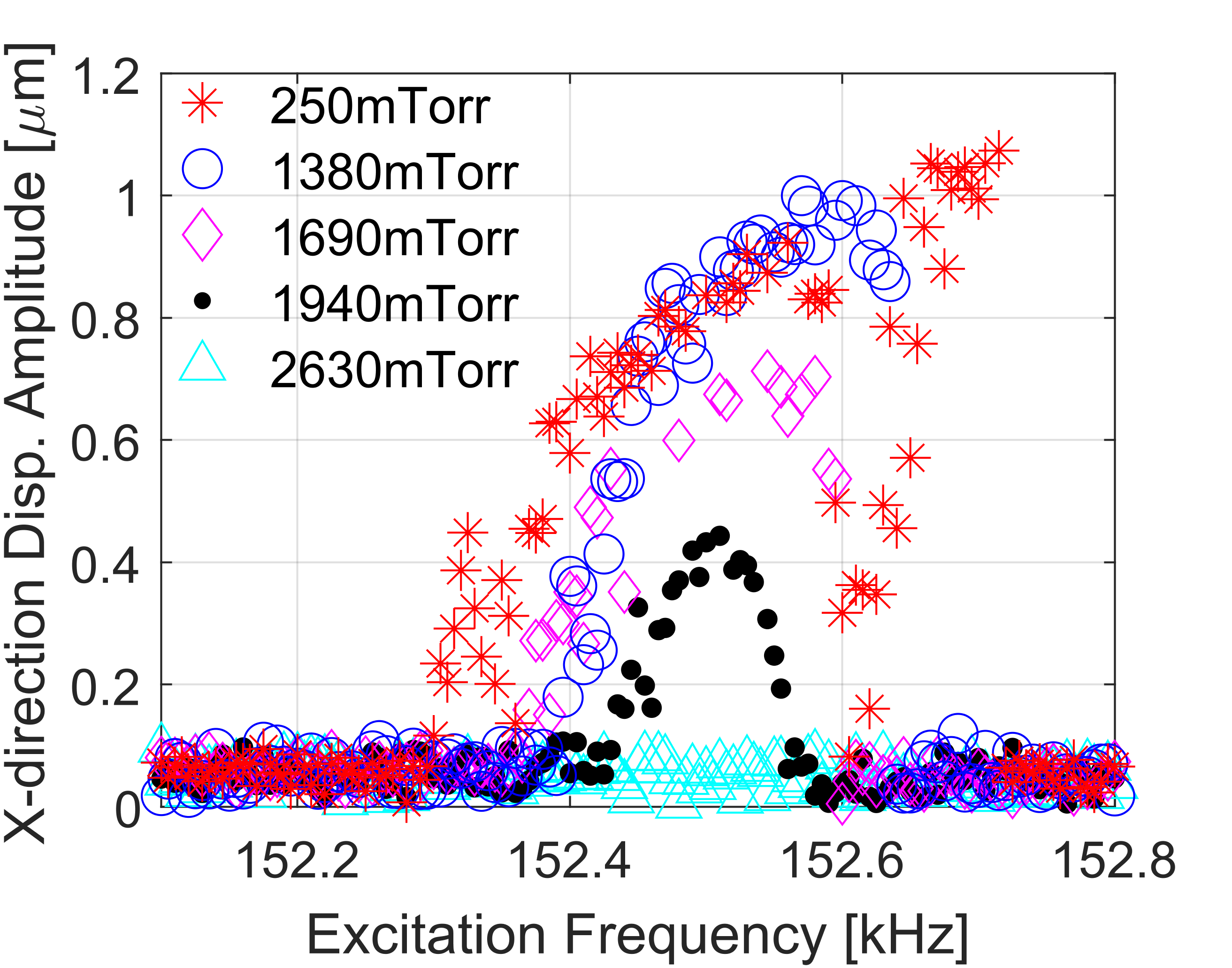

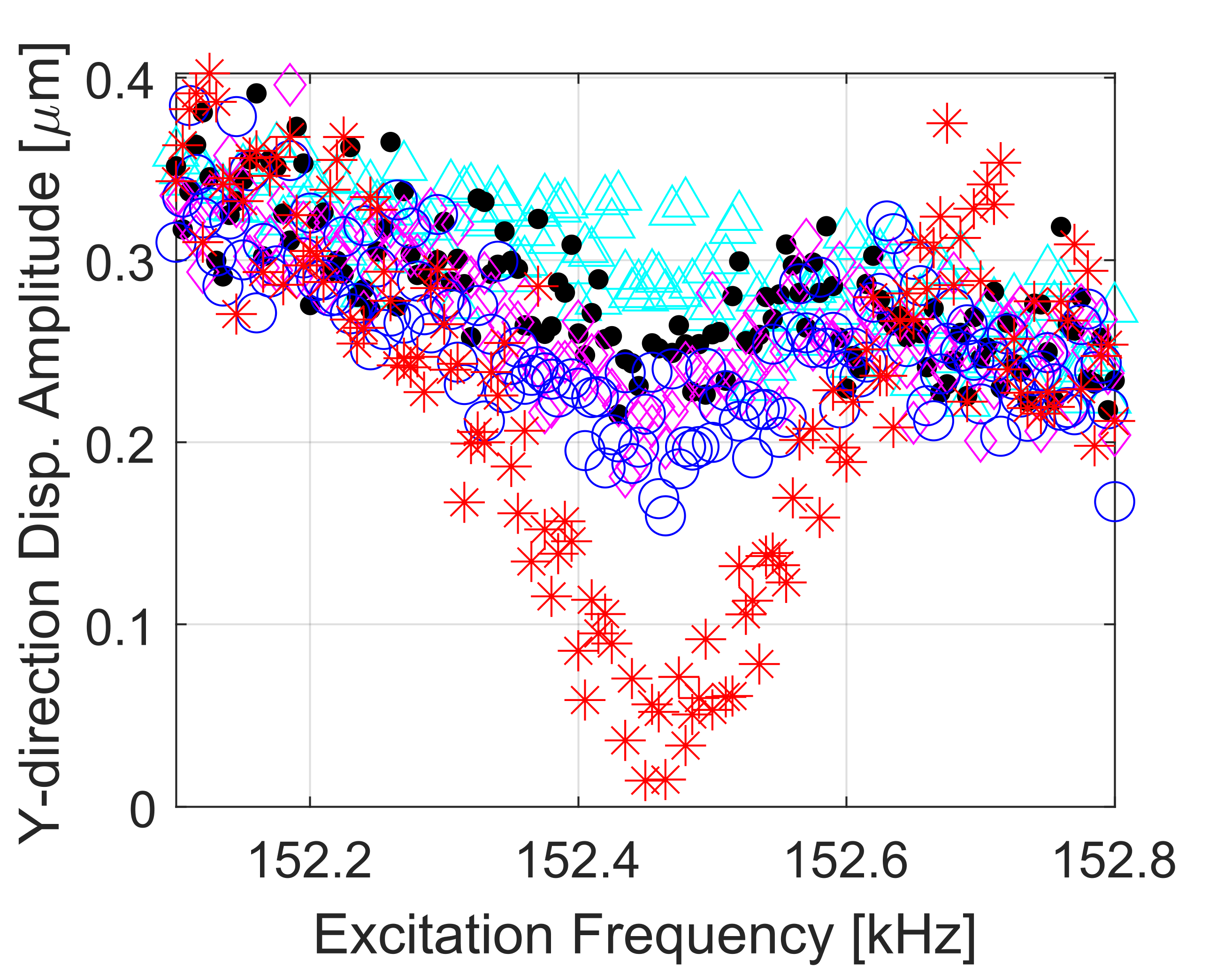


(a) (b)

Figure S4 – Experimental data of the frequency responses curves for different pressure values at V_DC2_ = 54V and V_AC2_ = 0.5V with E2 excitation around the 2^nd^ mode. (a) 1^st^ mode. (b) 2^nd^ mode. The frequency responses show that the saturation phenomenon endures high pressures. The frequency bandwidth that the phenomenon occurs reduces with the pressure increase and presents a linear response before the saturation diminishes.

# Parametric Study

Each term of Eqs. (1) and (2) has a dominance in the dynamics of the portal frame. Mainly, Fig. 5 shows that the displacement amplitude in the Y-direction of the supported beam increases by almost a factor of two while the V_AC2_ excitation slightly increases from 4.5V to 5.0V. The parameter responsible for this change is δ_2_, mainly the term . This term dominates the response of the portal frame when there is no internal resonance, and the V_DC2_ actuation is not high enough. Fig. S5 shows simulated frequency responses when δ_2_ is neglected (Fig. S5a), and accounting for δ_2_ (Fig. S5b), varying the AC voltage from the linear response (V_AC2_ = 4.5V) to the nonlinear one (V_AC2_ = 5.0V). Observe that, when δ_2_ = 0, the response tends to be of hardening type. In addition, when V_AC2_ > 4.7V, dynamic pull-in is reached. When δ_2_ is accounted for in the numerical simulations, note that a slight increase from 4.5V to 4.7V is enough to present the nonlinear softening behavior.


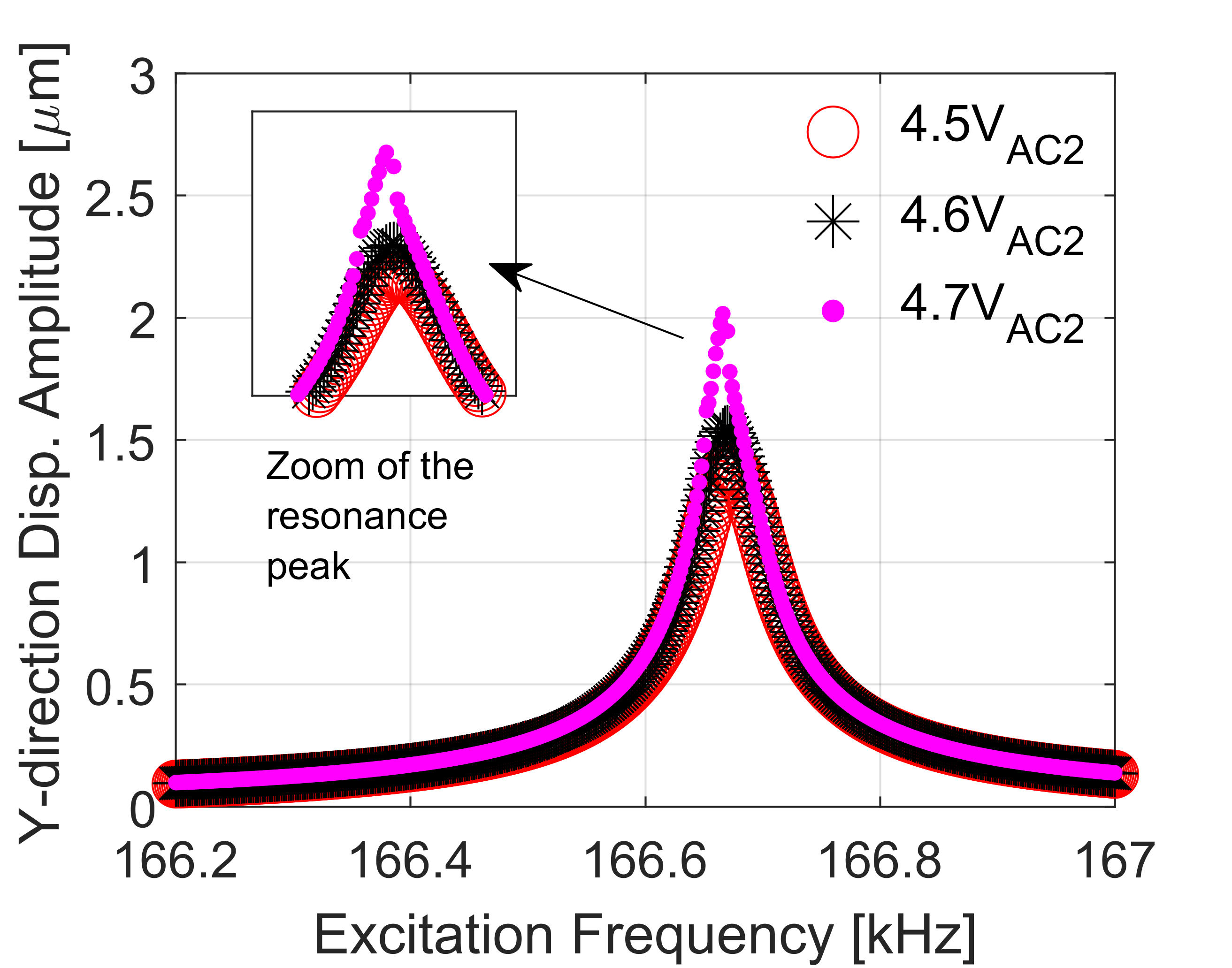

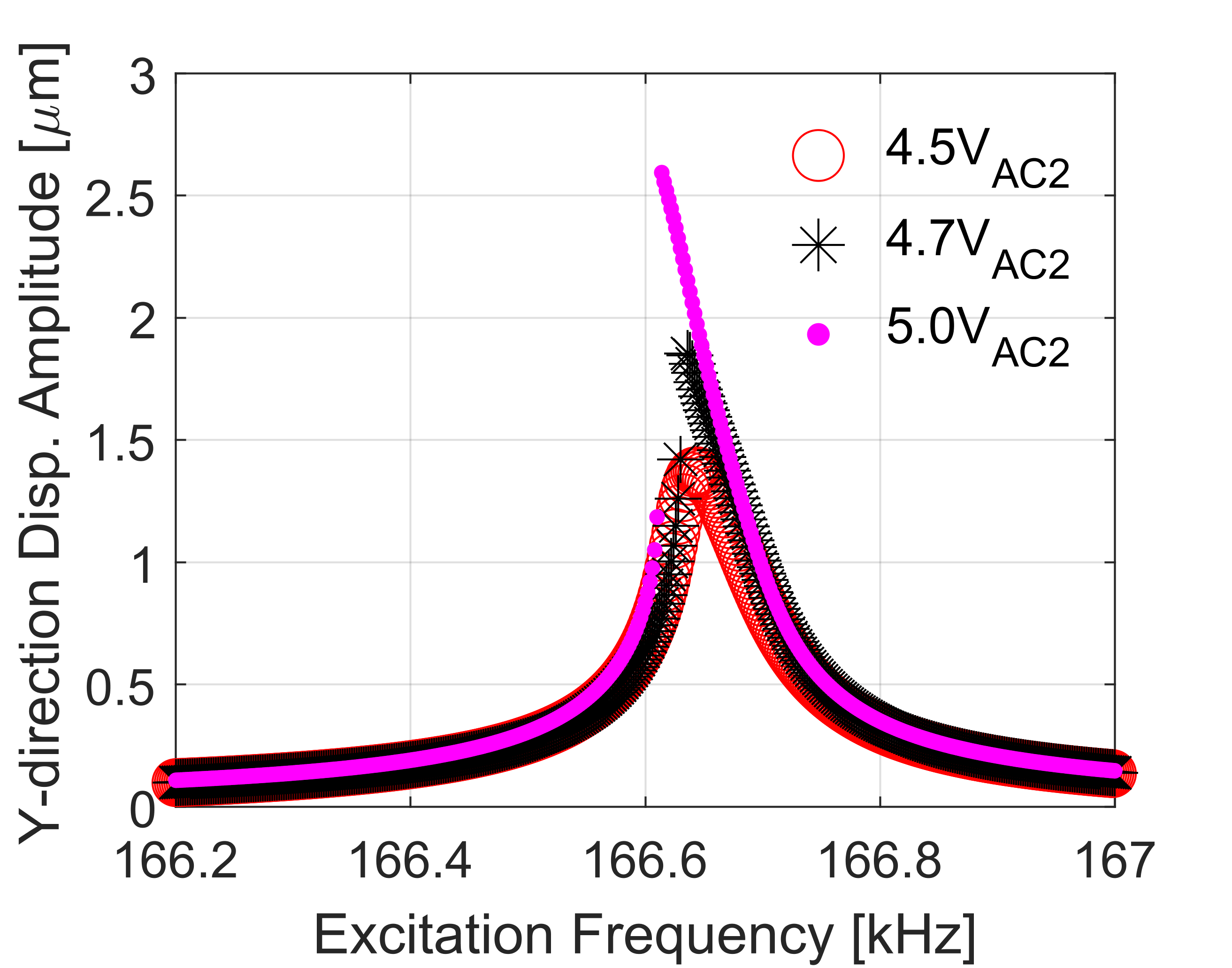


(a) (b)

Figure S5– Numerical simulations of the frequency responses of the 2^nd^ mode varying V_AC2_ in the interval [4.5, 5.0]V when (a) δ_2_ = 0, and (b) δ_2_ = δ_2_. The curves show that the displacement amplitude of the 2^nd^ mode increases almost a factor of two with a slight increase of V_AC2_ from 4.5V to 5.0V when δ_2_ is accounted for the numerical simulations. However, neglecting δ_2_, hardening behavior is observed.

In addition, depending on the value of δ_2_, the nonlinear behavior changes. Fig. S6 shows different frequency responses varying the parameter δ_2_ by magnitudes of the coefficient from 0 to 1 at V_AC2_ = 5.0V. Note that the smaller the coefficient, the softening behavior tends to become a hardening one, also indicated in Fig. S5a. Additionally, the softening behavior tends to be minimized to a linear response when the coefficient increases. However, if δ_2_ < 0.7δ_2_, dynamic pull-in is reached.


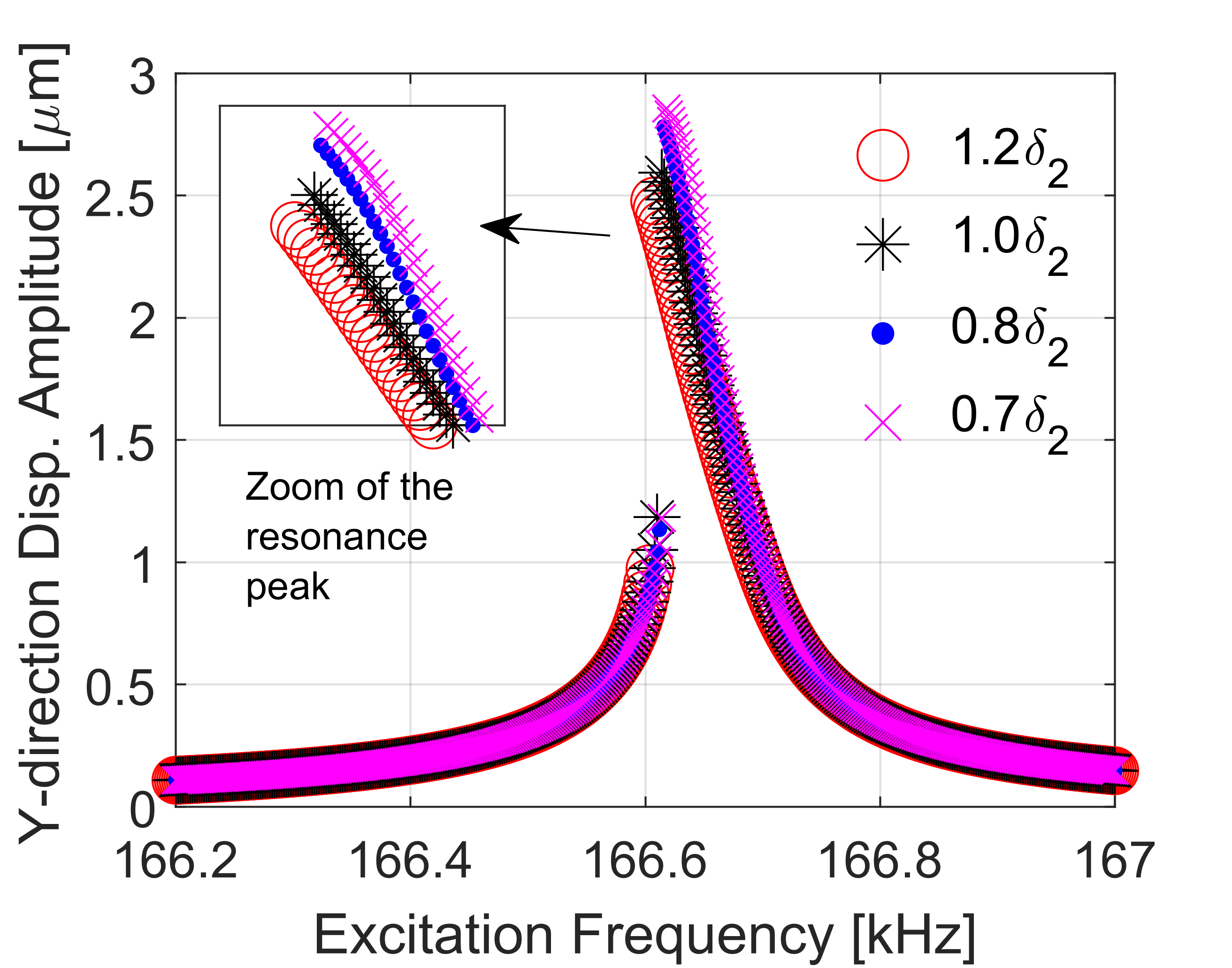


Figure S6 – Numerical simulations of the frequency responses of the 2^nd^ mode varying δ_2_ when V_AC2_ = 5.0V. The frequency responses show that the nonlinear softening behavior tends to become a hardening one as the coefficient δ_2_ decreases and can be minimized to a linear response when the coefficient increases.

However, the coefficients δ_1_ and δ_2_ are negligible for the internal resonance. Figs. S7 and S8 show the variation of δ_1_ and δ_2_, respectively, by magnitudes of the coefficients from 0 to 1 at V_DC2_ = 54V and V_AC2_ = 0.5V. Note that there is no change in the frequency response curves. We can also note that the 2^nd^ mode presents a nonlinear softening behavior around 149.8kHz, which also does not change as it is induced from the electrostatic force. Therefore, the internal resonance is only obtained due to the terms of the coefficients α_1_ and α_2_.


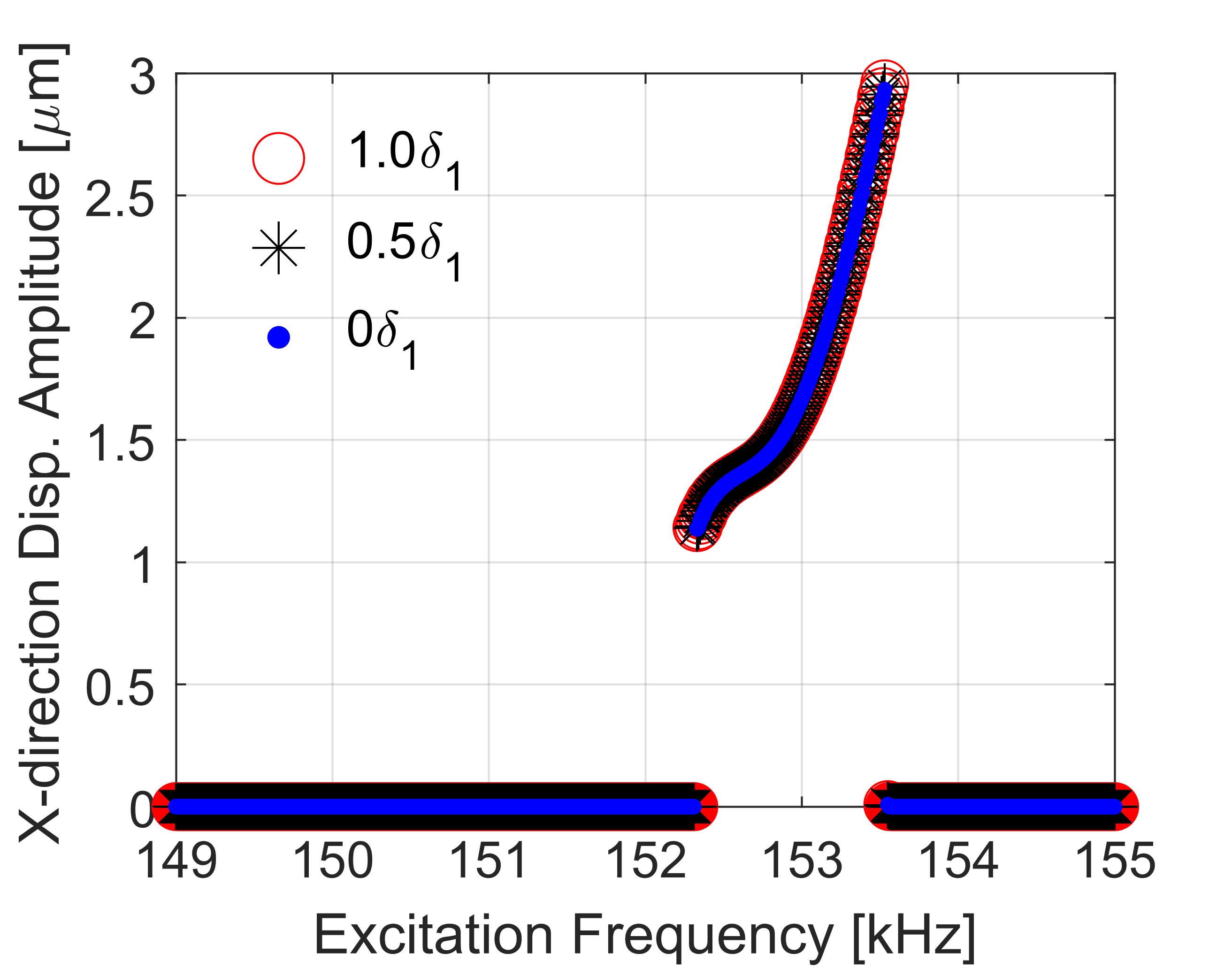

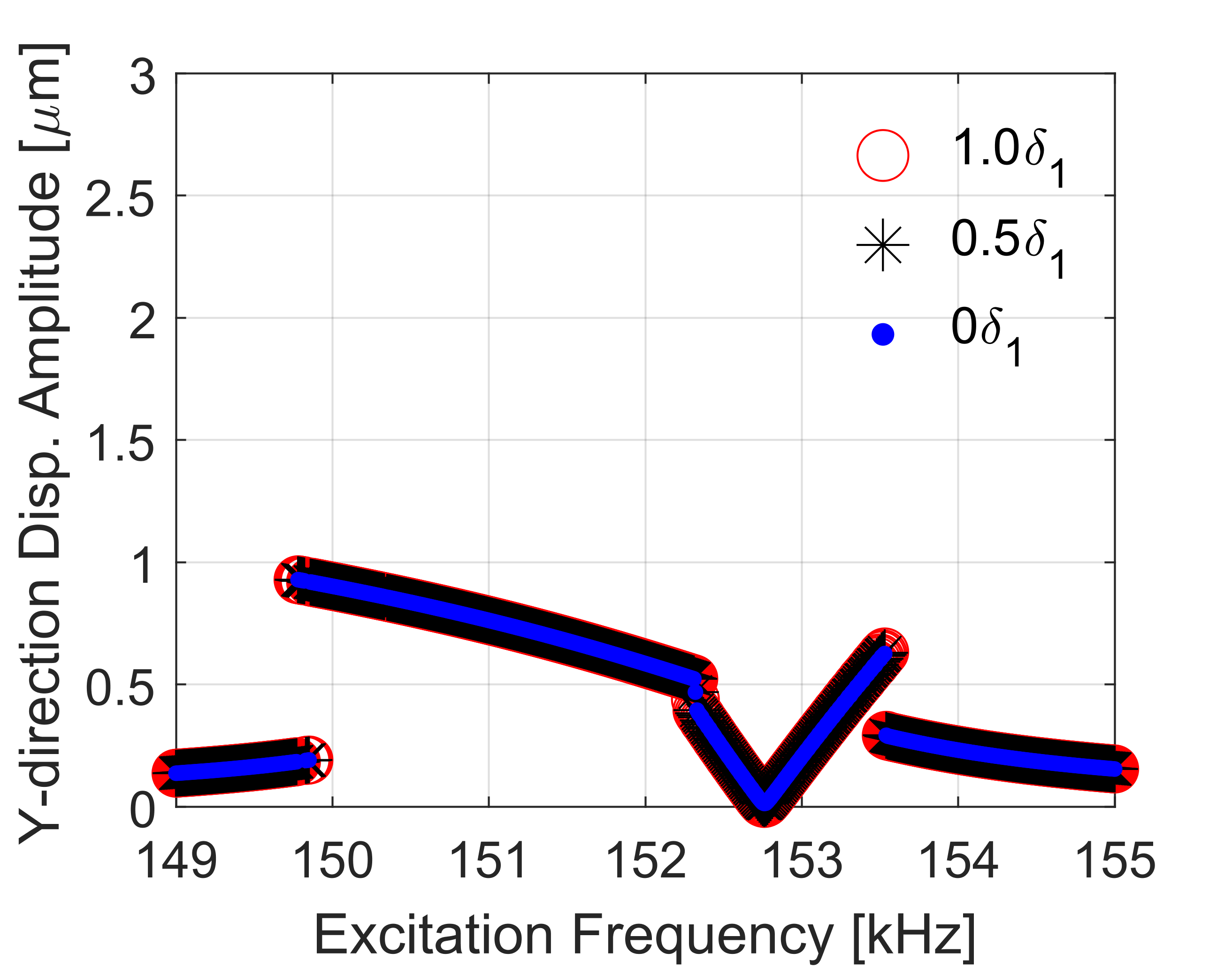


(a) (b)

Figure S7 – Numerical simulations of the frequency responses of (a) the 1^st^ mode, and (b) the 2^nd^ mode when varying the coefficient δ_1_ at V_DC2_ = 54V and V_AC2_ = 0.5V. The curves show that the coefficient δ_1_ is negligible for either the internal resonance or the nonlinear softening behavior induced by the electrostatic force.


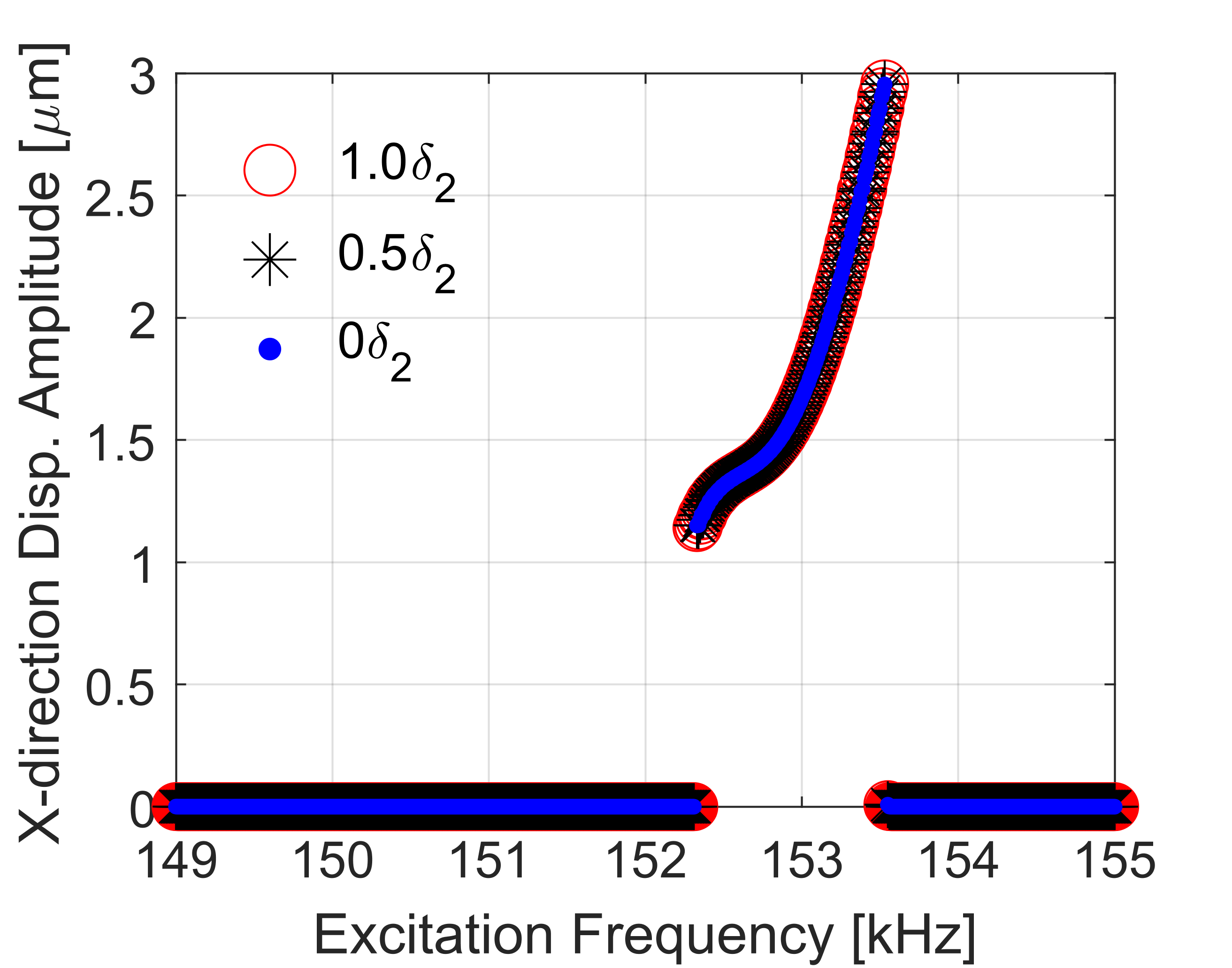

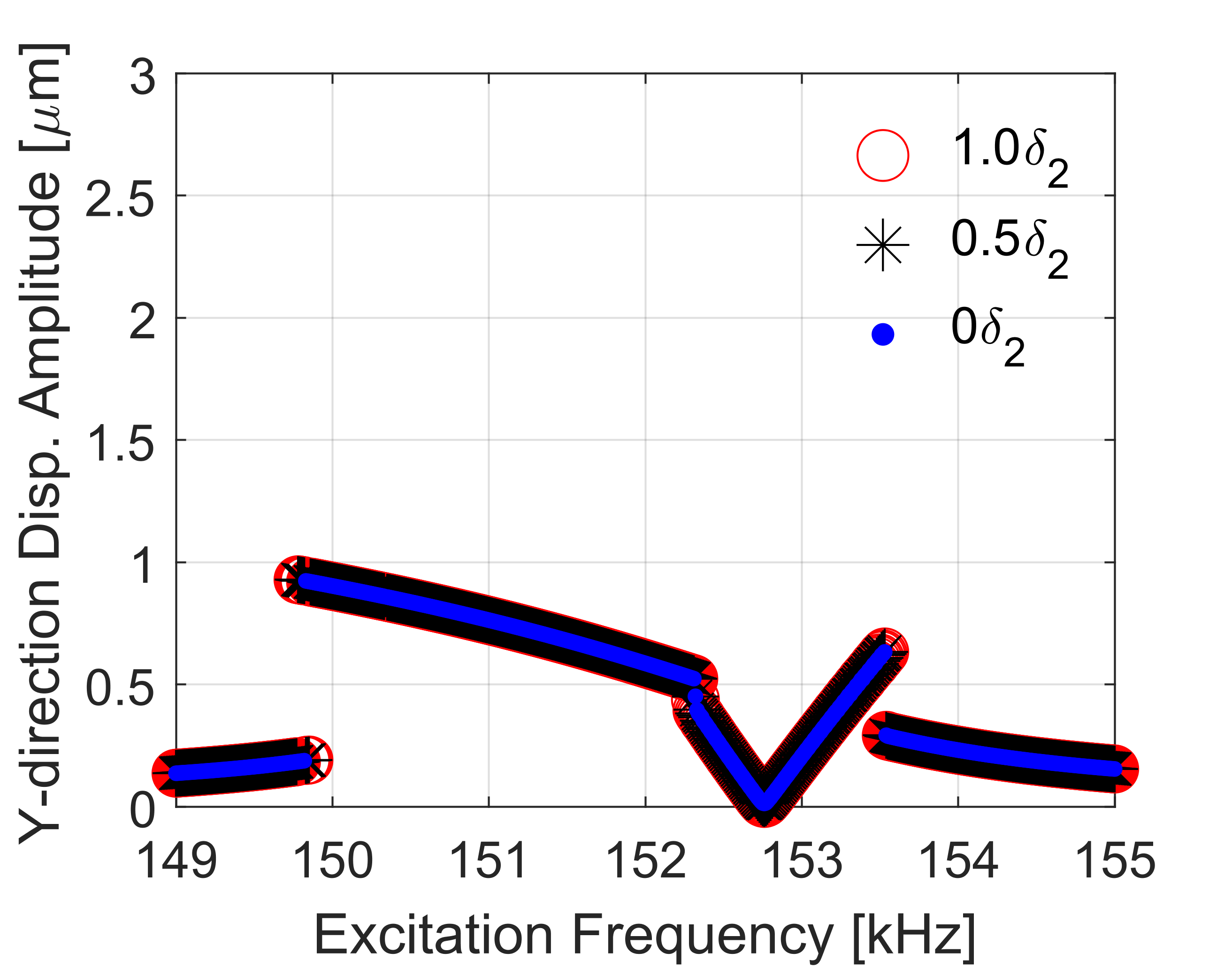


(a) (b)

Figure S8 – Numerical simulations of the frequency responses of (a) the 1^st^ mode, and (b) the 2^nd^ mode when varying the coefficient δ_2_ at V_DC2_ = 54V and V_AC2_ = 0.5V. The curves show that the coefficient δ_2_ is negligible for either the internal resonance or the nonlinear softening behavior induced by the electrostatic force.

# Mode Shape Developments

The portal frame structure of Fig. S1 can be modeled as a continuum system, as illustrated in Fig. S9. The continuum system has a reference general coordinate XOY_1_, and two others XOY_2_, and XOY_3_ rotated in relation to the reference one. Because of the symmetry of the structure, we model half of the portal frame, as in Fig. S10, divided on the mid-span of the supported beam to develop the equations of motion. Then, we model it as an Euler-Bernoulli L-shaped beam that undergoes only planar motion. Afterward, assuming symmetry between both sides, we impose boundary conditions that relate to both sides, considering the obtained ones from Hamilton’s principle.


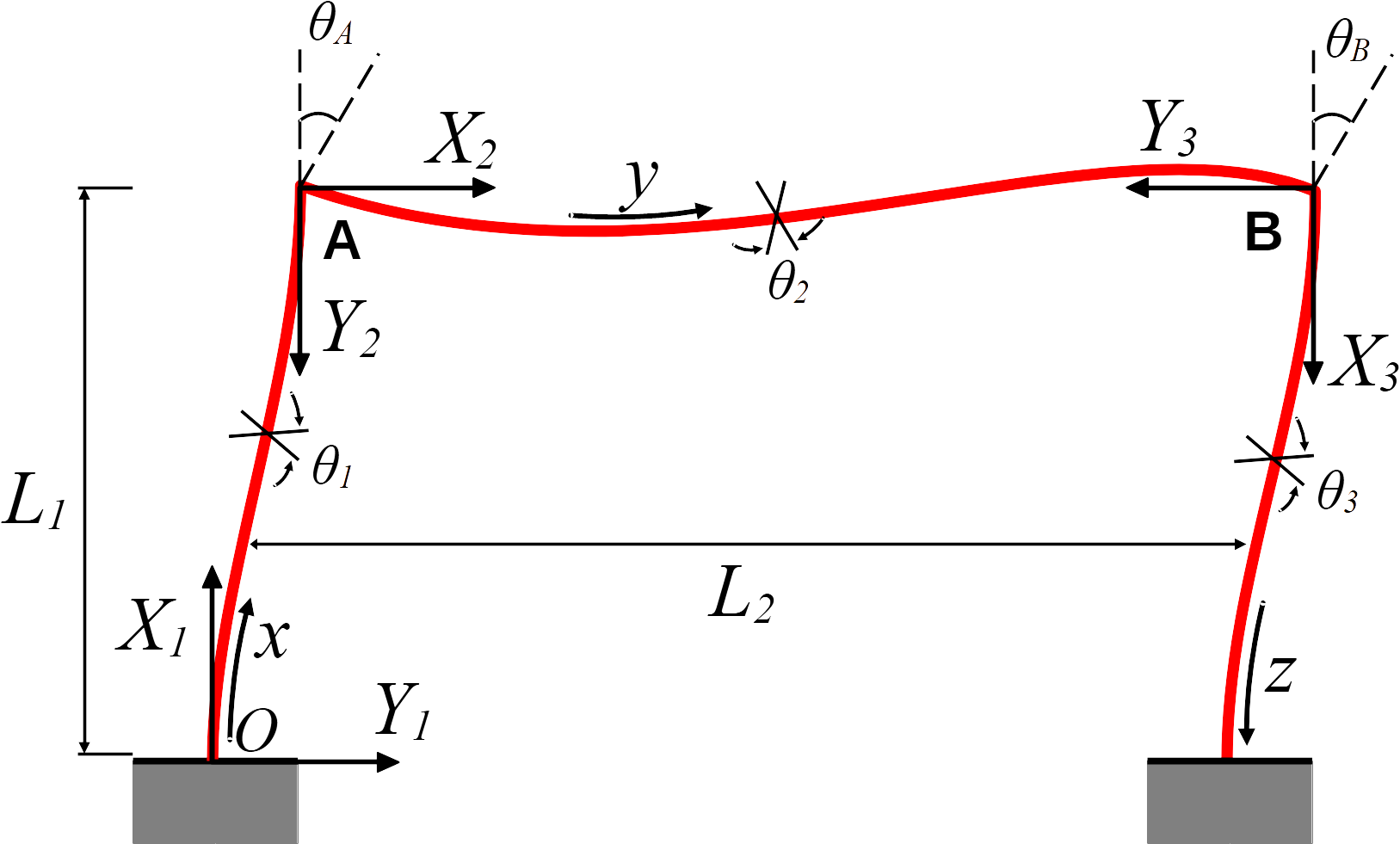


Figure S9 – Continuum portal frame structure schematic.


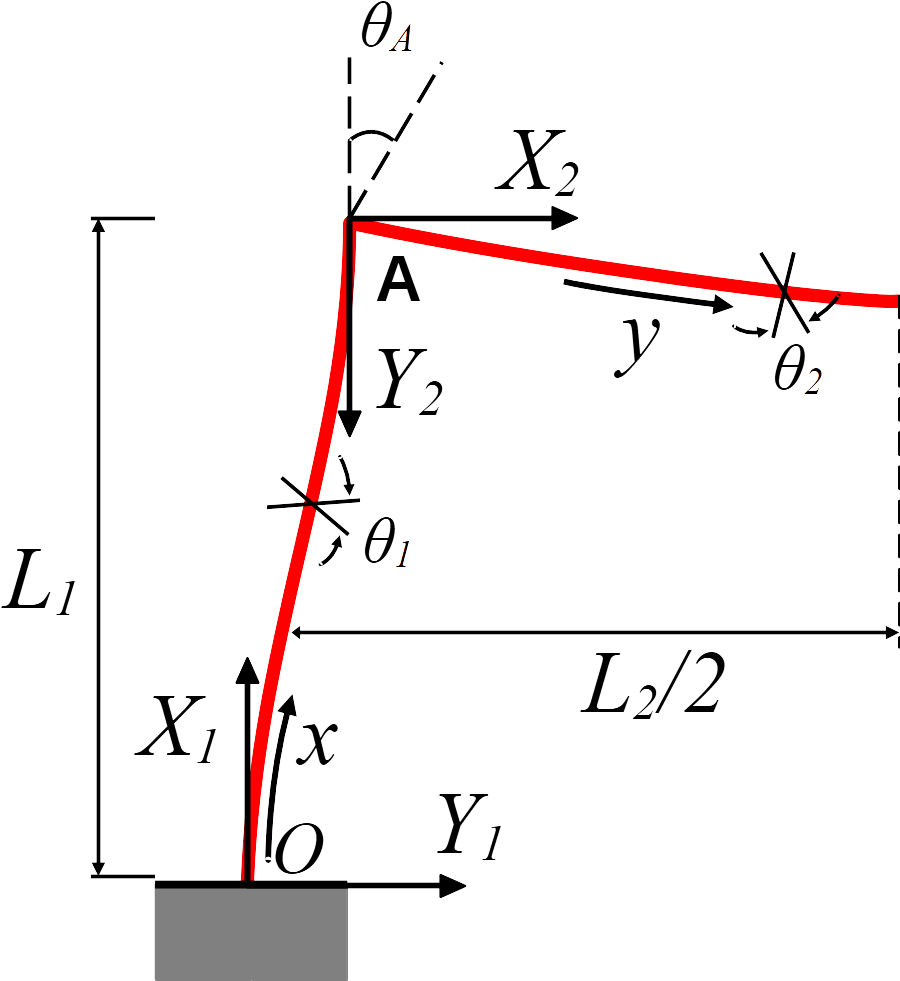


Figure S10– Left-hand side of the structure divided on the mid-span of the supported beam.

Each coordinate system is related to the axial and transversal displacements for each beam element as:

$\begin{matrix} X_{i}=u_{i}\left(s,t\right)+s \\ Y_{i}=w_{i}\left(s,t\right) \end{matrix}$ (S22)

where *u* and *w* are respectively the axial and transversal displacements, the index “*i*” is the beam element, and *s* is the independent spatial coordinate of each beam element. Note that *θ­*_1_ and *θ­*_2_ are the slopes of each beam element.

The kinetic energy *T* of the structure is given by

(S23)

where for convenience *L_b_* = *L_2_*/2, and *A*_c_ and *A_b_* are the cross-section areas of each beam element. The position vectors *r_1_* and *r_2_* of each beam motion are given by

$\begin{matrix} r_{1}=\left(u_{1}+x\right)i_{1}+w_{1}j_{1} \\ r_{2}=\left(u_{A}+x_{A}\right)i_{1}+w_{A}j_{1}+\left(u_{2}+y\right)i_{2}+w_{2}j_{2} \end{matrix}$ (S24)

where *i_1_* and *j_1_* are the reference unit vectors of XOY_1_, *i_2_* and *j_2_* are the reference unit vectors of XOY_2_, and the subscript *A* is the intersection between the beam elements with respect to *L_1_*, read as

$\begin{matrix} u_{A}=u_{1}\left(x,t\right)|_{x=L_{1}} \\ w_{A}=w_{1}\left(x,t\right)|_{x=L_{1}} \\ x_{A}=L_{1} \end{matrix}$ (S25)

Due to the reference XOY rotating with the cross-section bend angle of the column beam, the axis *i* and *j* can rotate according to

$\left\{\begin{matrix} i_{2} \\ j_{2} \end{matrix}\right\}=\left[\begin{matrix} -\sin\theta_{A} & \cos\theta_{A} \\ -\cos\theta_{A} & -\sin\theta_{A} \end{matrix}\right]\left\{\begin{matrix} i_{1} \\ j_{1} \end{matrix}\right\}$ (S26)

where $\sin\theta_{A}=w'_{A}$ and $\cos\theta_{A}=1+u'_{A}$. Note that to obtain the rotation relation of Eq. (S26), an angle of 90-degrees is imposed between the XOY_1_ and XOY_2_. In addition, due to the inextensibility of the elements, we have

$(1+u'_{i})^{2}+w'_{i}^{2}=1$ (S27)

where $\sin\theta_{i}=w'_{i}$ and $\cos\theta_{i}=1+u'_{i}$. Since the deflections of the L-shaped beam in each section are considered small, we have the following geometrical relationships

$\begin{matrix} \cos\theta_{i}=1+u'_{i}=\sqrt{1-w'_{i}^{2}}=1+O\left(\varepsilon^{2}\right) \\ \theta_{i}=\arcsin w'_{i}=w'_{i}+O\left(\varepsilon^{3}\right) \end{matrix}$ (S28)

Introducing Eqs. (S26) into Eq. (S24), taking the derivative with respect to time and multiplication, we obtain

(S29)

where we neglected terms of cubic order.

The total potential energy of the structure is given by

$U=\frac{1}{2}EI_{c}\int_{0}^{L_{1}} \theta'_{1}^{2}dx+\frac{1}{2}EI_{b}\int_{0}^{L_{b}} \theta'_{2}^{2}dy$ (S30)

Applying Hamilton’s principle with Eqs. (S23) and (S30), neglecting damping and terms above cubic order, the equations of motions of the half portal-frame (L-shaped beam) in the strong form are given by

(S31)

where the boundary conditions are obtained as

(S32)

Introducing a change of variable for $W_{2}=w_{2}+yw'_{A}$, the equations of motion become

(S33)

In addition, substituting *W_2_* into the BCs of Eq. (S32) and assuming from the linear free vibration of the 2^nd^ equation of Eq. (S33) that , the boundary conditions can be rewritten as

(S34)

Now, admitting the entire supported beam element (*L_b_* = *L_2_*) with the column on the right-hand side of the structure and imposing symmetry of both sides, we have the complete portal frame as shown in Fig. S9. Note that the coordinates at joint B are rotated to be the inverse of joint A. Rotations and bending are maintained. However, the shear force direction is inverted. These assumptions result in the BCs as below

(S35)

where the free vibration equations of motion can be written as

(S36)

Carrying out a non-dimensional procedure with the new non-dimensional variables as below

$$\hat{w}_{1}=\frac{w_{1}}{L_{1}}\text{; }\hat{w}_{2}=\frac{W_{2}}{L_{2}}\text{; }\hat{w}_{3}=\frac{w_{3}}{L_{1}}\text{; }\hat{x}=\frac{x}{L_{1}}\text{; }\hat{y}=\frac{y}{L_{2}}\text{; }\hat{z}=\frac{z}{L_{1}}\text{; }\hat{t}=\frac{t}{T}\text{; }T=\sqrt{\frac{\rho A_{c}L_{1}^{4}}{EI_{c}}};$$

and that *L_3_* = *L_1_*, the equations of motion and boundary conditions finally become, respectively

(S37)

(S38)

## Supplementary References

[S1] Palacios, J. L., Balthazar, J. M., & Brasil, R. M. L. R. F. On non-ideal and nonlinear portal frame dynamics analysis using Bogoliubov averaging method. Journal of the Brazilian Society of Mechanical Sciences, 24(4), 257-265 (2002).

# Supplementary Videos

Video 1 – Animation of the portal frame under 2:1 internal resonance and saturation phenomenon.

This video shows the animation of the portal frame under the activation of 2:1 internal resonance alongside the simulated time histories of displacements of both modes. In this animation, the 2^nd^ mode is excited around the resonance (at 152.9 kHz) with 0.5V_AC2_ at 54V_DC2_. The transient response is observed from 0 to 220s, where the 2^nd^ mode response increases. After 220s, the 2^nd^ mode saturates and the 1^st^ mode is activated, becoming higher than the 2^nd^ one. Then, after 900s approximately, the steady-state response is reached. Long time is seen due to the non-dimensional time.

Video 2 – Experimental video of the steady-state response obtained after the saturation phenomenon.

The video of the portal frame is recorded at one oscillation period excited through electrode 2 with 0.5V_AC2_ at 54V_DC2_ and 152.2 kHz. In addition, the camera is synchronized with ½ of the frequency of excitation to, therefore, the 1^st^ mode can be seen.
